# Interface Engineering of Co/CoMoN/NF Heterostructures for High‐Performance Electrochemical Overall Water Splitting

**DOI:** 10.1002/advs.202105313

**Published:** 2022-02-11

**Authors:** Haibin Ma, Zhiwen Chen, Zhili Wang, Chandra Veer Singh, Qing Jiang

**Affiliations:** ^1^ Key Laboratory of Automobile Materials Ministry of Education, and School of Materials Science and Engineering Jilin University Changchun 130022 China; ^2^ Department of Materials Science and Engineering University of Toronto 184 College Street, Suite 140 Toronto ON M5S 3E4 Canada; ^3^ Department of Mechanical and Industrial Engineering University of Toronto 5 King's College Road Toronto ON M5S 3G8 Canada

**Keywords:** heterostructures, hydrogen evolution reaction, interface engineering, overall water splitting, oxygen evolution reaction

## Abstract

The development of low‐cost and high‐efficiency catalysts for both hydrogen evolution reaction (HER) and oxygen evolution reaction (OER) in alkaline electrolyte is still challenging. Herein, interfacial Co/CoMoN heterostructures supported on Ni foam (Co/CoMoN/NF) are constructed by thermal ammonolysis of CoMoO*_x_*. In 1.0 m KOH solution, Co/CoMoN/NF heterostructures exhibit excellent HER activity with an overpotential of 173 mV at 100 mA cm^−2^ and a Tafel slope of 68.9 mV dec^−1^. Density functional theory calculations indicate that the low valence state Co site acts as efficient water‐dissociation promoter, while CoMoN substrate has favorable hydrogen adsorption energy, leading to an enhanced HER activity. The Co/CoMoN/NF heterostructures also achieve high OER activity with an overpotential of 303 mV at 100 mA cm^−2^ and a Tafel slope of 56 mV dec^−1^. Using Co/CoMoN/NF heterostructures as the cathode and anode, the alkaline electrolyzer requires a low voltage of 1.56 V to reach the current density of 100 mA cm^−2^ along with superior long‐term durability. This study provides a new design strategy toward low‐cost and excellent catalysts for water splitting.

## Graphical abstract

Co/CoMoN/NF exhibits excellent electrochemical water splitting performance in alkaline media via construction of interfacial heterostructures.

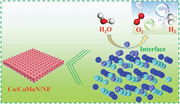

## 1 Introduction

Hydrogen has been regarded as a highly promising candidate to replace the traditional fossil fuels.^[^ ^1^, ^2^ ^]^ Among various hydrogen production techniques, electrochemical water splitting is one of the most efficient, clean, and safe routes to generate hydrogen.^[^ ^3^ ^]^ Alkaline water splitting is more suitable for the commercial application, but it still suffers from low efficiency,^[^ ^4^ ^]^ high operation overpotential,^[^ ^5^, ^6^, ^7^ ^]^ and poor durability.^[^ ^8^ ^]^ Such dilemma motivates researchers to explore highly efficient catalyst for hydrogen evolution reaction (HER) and oxygen evolution reaction (OER).^[^ ^9^ ^]^ To date, noble metal Pt‐based materials^[^ ^10^, ^11^ ^]^ and Ir/Ru oxides such as IrO_2_^[^ ^12^ ^]^ and RuO_2_^[^ ^13^ ^]^ are regarded as the most efficient catalysts for HER and OER, respectively. In view of the high‐cost and low earth‐abundance of these noble metals, much effort have been focused on developing new catalysts based on non‐noble transition metals such as Ni, Fe, Co, and Mo^[^ ^14^ ^]^ to simultaneously improve HER and OER performances. Nevertheless, the water splitting performance of precious metal‐free catalysts in alkaline media is still low due to the sluggish water dissociation kinetics in the Volmer step^[^ ^15^, ^16^, ^17^ ^]^ and unfavorable OH^−^ adsorption energetics.^[^ ^18^ ^]^

An efficient strategy to simultaneously enhance alkaline HER and OER performances of catalyst is to build an appropriate interface that introduces a water dissociation promoter to cleave H—OH bonds to accelerate the Volmer step of HER^[^ ^15^, ^19^ ^]^ and an OH^−^ adsorption promoter to accelerate the kinetics of OER.^[^ ^18^, ^20^ ^]^ Previous studies demonstrate that transition metal nanoparticles (NPs) such as Ni^[^ ^21^, ^22^ ^]^ are promising water dissociation promoters for HER, with which highly reactive d electrons of transition metal can efficiently cleave H—OH bonds.^[^ ^23^ ^]^ Transition metal oxides/hydroxides such as CoOOH,^[^ ^24^ ^]^ NiOOH,^[^ ^25^, ^26^ ^]^ and Co_3_O_4_^[^ ^27^ ^]^ have beneficial OH^−^ adsorption energies for OER. However, the limited electronic conductivity of these oxides/hydroxides hinders electron transfer at high current density,^[^ ^28^ ^]^ which limits their OER activity. Transition metal nitrides have higher electronic conductivity property than transition metal oxides/hydroxides. Moreover, transition metal nitrides such as MoN have favorable H* adsorption energetics^[^ ^21^, ^22^, ^29^ ^]^ for HER. Based on the dual‐descriptor method,^[^ ^30^ ^]^ the reasonable design of interfacial synergetic heterostructures that consists of metal NPs and transition metal nitrides could be a candidate to enhance HER and OER simultaneously in alkaline media.^[^ ^31^ ^]^

On the basis of the above considerations, the charge polarized Co/CoMoN heterostructures supported on Ni foam (Co/CoMoN/NF) were constructed for water splitting in alkaline media. The Co/CoMoN/NF heterostructures show superior HER activity with an overpotential of 173 mV at 100 mA cm^−2^ and a Tafel slope of 68.9 mV dec^−1^. Density functional theory (DFT) calculations demonstrate that the interfacial effect between Co NPs and CoMoN nanosheets leads to significantly enhanced HER kinetics. Moreover, the Co/CoMoN/NF heterostructures also show excellent OER activity with an overpotential of 303 mV at 100 mA cm^−2^ and a Tafel slope of 56 mV dec^−1^. Spontaneously, Co/CoMoN/NF heterostructures can be utilized as both cathode and anode in full water splitting, which is enabled to take place at an extremely low voltage of 1.56 V to drive current density of 100 mA cm^−2^ along with superior durability in alkaline media.

## 2 Results and Discussion

**Figure 1:**
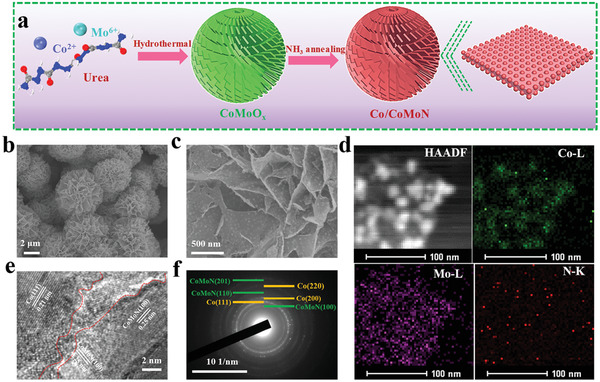
a) Schematic illustration of the fabrication for hierarchical Co/CoMoN/NF flower‐like microspheres consisting of many aligned nanosheets. b,c) SEM images of Co/CoMoN/NF. d) TEM mapping of Co/CoMoN/NF. e) HRTEM image of Co/CoMoN/NF. f) SAED image of Co/CoMoN/NF.

A two‐step method is developed to prepare Co/CoMoN/NF heterostructures, as illustrated in **Figure** **1**a. In the first step, the CoMoO*_x_* grown on Ni foam (NF) was synthesized using a facial hydrothermal method, in which a homogenous solution of Co(NO_3_)_2_·6H_2_O and (NH_4_)_6_Mo_7_O_24_·4H_2_O was heated at 120 °C for 12 h in a 100 mL Teflon‐lined autoclave. Scanning electron microscopy (SEM) images of CoMoO*_x_*/NF show that the product of CoMoO*_x_* has a flower‐like microsphere structure assembled from thin nanosheets (Figure S1, Supporting Information). This is in agreement with previous studies that indicate that the utilization of urea and NH_4_F in the hydrothermal procedure can produce flower‐like microsphere structure due to thermodynamic preferences.^[^ ^32^, ^33^ ^]^ Much more detailed experimental and theoretical studies are needed to elucidate the exact formation mechanism of flower‐like microsphere structure. X‐ray photoelectron spectroscopy (XPS) results reveal that the Co and Mo elements are in the oxidized state (Figure S2, Supporting Information). Subsequently, the obtained CoMoO*_x_*/NF sample was annealed under ammonia atmosphere at 500 °C for 2 h with a ramp rate of 2 °C min^−1^. After this thermal ammonolysis reaction step, the Co/CoMoN/NF heterostructures were obtained. For comparison, CoMoN/NF electrode without Co NPs decoration was prepared by etching the Co NPs of Co/CoMoN/NF heterostructures by HCl solution (Figure S3, Supporting Information). Metallic Co particles grown on NF (Co/NF) was also prepared via the similar method as Co/CoMoN/NF except the (NH_4_)_6_Mo_7_O_24_·4H_2_O was not used as metal precursor (Figure S4, Supporting Information).

**Figure 2:**
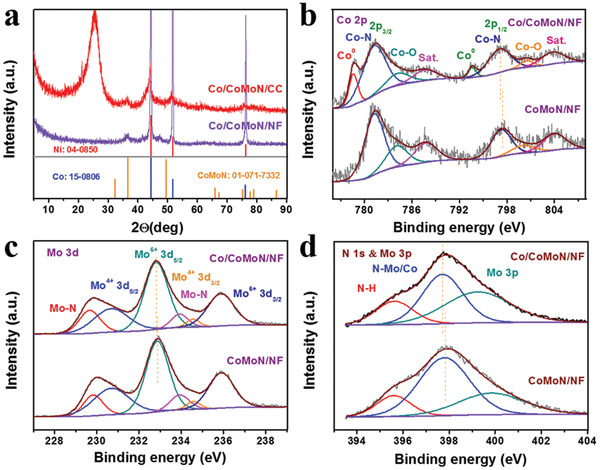
a) XRD patterns of as‐prepared Co/CoMoN/NF and Co/CoMoN/CC. High‐resolution XPS spectra of b) Co 2p, c) Mo 3d, d) N 1s, and Mo 3p for Co/CoMoN/NF and CoMoN/NF.

The micro‐nanostructures of as‐synthesized samples were characterized by SEM and transmission electron microscopy (TEM). Low‐magnification (Figure S5, Supporting Information) and high‐magnification (Figure 1b) SEM images of the as‐synthesized Co/CoMoN/NF heterostructures reveal that the flower‐like nanosheets microspheres with an average diameter of 4.65 µm are well grown on the skeleton of NF. Figure 1c shows a typical TEM image of the Co/CoMoN composite scratched off from the Co/CoMoN/NF heterostructures. It can be seen that the nanosheet was homogeneously decorated with a large number of NPs with an average diameter of 15.6 nm (Figure S6, Supporting Information), which can provide abundant interfacial sites for electrocatalytic reactions. The high‐angle annular dark‐field scanning TEM and corresponding elemental mapping images (Figure 1d) show that Co is mainly concentrated in the NPs, whereas Mo and N are distributed more homogeneously, indicating that the NPs and nanosheets were composed of Co and CoMoN, respectively. The high resolution TEM image of Co/CoMoN/NF heterostructures (Figure 1e) reveals the well‐resolved lattice fringes with the lattice distances of 0.25 and 0.21 nm, which are consistent with that of the (100) plane of Co_0.2_Mo_0.8_N and (111) plane of Co, respectively. The selected areas electron diffraction pattern (Figure 1f) further confirms the existence of Co_0.2_Mo_0.8_N and metallic Co phases. The crystal structure of Co/CoMoN/NF heterostructures was further confirmed by X‐ray diffraction (XRD). It is difficult to observe the diffraction peaks of Co NPs from the XRD pattern of Co/CoMoN/NF because the diffraction peaks corresponding to Co NPs are covered by that of NF substrate (**Figure** **2**a). To get rid of the influence of NF substrate patterns, Co/CoMoN grown on carbon cloth (Co/CoMoN/CC) was synthesized for XRD measurement. As show in Figure 2a, the diffraction peaks located at 36.5°, 49.4°, 75.0°, and 77.6° are indexed to Co_0.2_Mo_0.8_N (JCPDS No. 01‐071‐7332), and the diffraction peaks located at 44.2°, 51.5°, and 75.9° are associated with the (111), (200), and (220) planes of metallic Co (JCPDS No. 15‐0806), respectively, further confirming the coexistence of CoMoN and metallic Co phases in Co/CoMoN/NF heterostructures.

The surface composition and chemical states of elements in as‐prepared samples were investigated by XPS shown in Figure 2b–d. The XPS survey spectra demonstrate the presence of Co, Mo, and N elements, and the Co/Mo/N atomic ratios on the surface of Co/CoMoN/NF and CoMoN/NF are calculated to be 1.0/2.7/7.4 and 1.0/2.8/8.9, respectively (Figure S7, Supporting Information). The atomic ratio of Co/Mo in the bulk Co/CoMoN/NF and CoMoN/NF heterostructures were measured by ICP‐OES to be 1.22/1 and 0.28/1, respectively, indicating the existence of Co_0.2_Mo_0.8_N phase in these samples, which is consistent with HRTEM and XRD results. As for Co/CoMoN/NF heterostructures, two peaks with the binding energies of 778.6 and 793.7 eV are observed in the Co 2p spectrum (Figure 2b), which corresponds to the Co 2p_3/2_ and Co 2p_1/2_ of metallic Co^0^,^[^ ^31^, ^34^ ^]^ indicating the presence of Co^0^ NPs. Two peaks at 781.4 and 797.2 eV can be ascribed to the Co—N bonds^[^ ^35^, ^36^ ^]^ of CoMoN phase. Another two peaks located at 784.4 and 800.6 eV are the Co^2+^ 2p_3/2_ and Co^2+^ 2p_1/2_ due to surface oxidation.^[^ ^37^, ^38^ ^]^ For Mo 3d spectrum (Figure 2c), two peaks at 229.7 and 233.9 eV can be attributed to Mo—N bonds^[^ ^35^ ^]^ of CoMoN phase. The peaks with the binding energies at 230.7 and 232.8, and 234.6 and 235.9 eV can be attributed to Mo^4+^ and Mo^6+^, respectively,^[^ ^21^, ^37^, ^39^, ^40^ ^]^ suggesting the existence of a small amount of Mo oxides such as MoO_2_ and MoO_3_ on the surface of Co/CoMoN/NF heterostructures due to surface oxidation. The Mo 3p and N 1s spectrum (Figure 2d) shows three peaks at 399.4, 397.7, and 395.6 eV, corresponding to Mo 3p_3/2_,^[^ ^21^, ^35^ ^]^ N—Mo/Co,^[^ ^35^, ^39^ ^]^ and N—H^[^ ^21^ ^]^ bonds, respectively. For CoMoN/NF electrode, the peaks corresponded to the metallic Co^0^ disappear, confirming that the Co NPs were removed after acid treatment. Surprisingly, the Co—N, Mo^6+^, Mo—N, and N 1s peaks in Co/CoMoN/NF shift to higher binding energy states by 0.32, 0.10, and 0.13 eV, respectively, compared with the CoMoN/NF, indicating the interfacial interactions between Co NPs and CoMoN are strong, leading to some electrons transfer from the decorated Co NPs to CoMoN through the Co/CoMoN interfaces.

**Figure 3:**
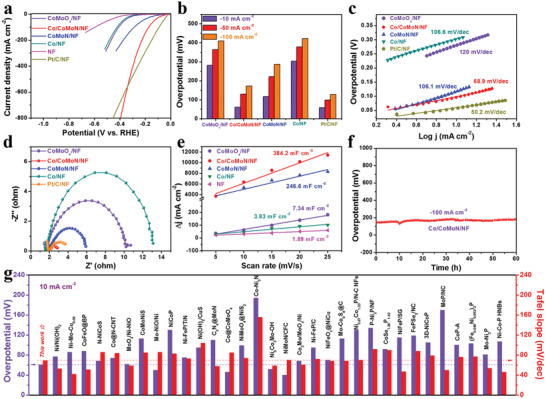
a) Typical polarization curves of CoMoO*_x_*/NF, Co/CoMoN/NF, CoMoN/NF, Co/NF, NF, and Pt/C/NF, sweep rate: 10 mV s^−1^, electrolyte: 1.0 m KOH. b) Overpotential at current density of −10, −50, and −100 mA cm^–2^ for CoMoO*_x_*/NF, Co/CoMoN/NF, CoMoN/NF, Co/NF, and Pt/C/NF. c) Tafel plots of different catalysts. d) EIS spectra of CoMoO*_x_*/NF, Co/CoMoN/NF, CoMoN/NF, Co/NF, and Pt/C/NF. e) Charging current density differences plotted against scan rates of CoMoO*_x_*/NF, Co/CoMoN/NF, CoMoN/NF, Co/NF, NF, and Pt/C/NF; *C*_edl_ is equivalent to the slope of fitted line. f) Stability test of Co/CoMoN/NF at current density of −100 mA cm^−2^ for 60 h in 1.0 m KOH. g) Tafel slope and overpotential at −10 mA cm^−2^ of Co/CoMoN/NF, comparing with the values of representative HER catalysts reported previously.

The HER performances of Co/CoMoN/NF, CoMoN/NF, CoMoO*_x_*/NF, Co/NF, NF, and 20 wt% Pt/C supported on NF (Pt/C/NF) electrodes were evaluated in 1.0 m KOH solution. The Hg/HgO reference electrode was calibrated against the reversible hydrogen electrode (RHE) (Figure S8, Supporting Information). **Figure** **3**a shows the polarization curves of different electrodes at a scan rate of 10 mV s^−1^ with 85% iR compensation.^[^ ^9^, ^41^ ^]^ It can be seen that the Co/CoMoN/NF heterostructures exhibit much better HER activity than CoMoN/NF, CoMoO*_x_*/NF, Co/NF, and NF electrodes. The Co/CoMoN/NF heterostructures require small overpotentials of 61 and 173 mV to reach the current densities of 10 and 100 mA cm^−2^ (Figure 3b), respectively, which are much lower than those of the CoMoN/NF (117 and 287 mV), CoMoO*_x_*/NF (281 and 410 mV), and Co/NF (304 and 422 mV) electrodes. Although the overpotential to achieve current density of 10 mA cm^−2^ is higher than that of Pt/C/NF electrode, the Co/CoMoN/NF heterostructures exhibit much higher HER activity than Pt/C/NF electrode at large current densities (>560 mA cm^−2^), and deliver the Faradaic efficiencies of H_2_ above 95% at overpotentials of 400, 500, and 600 mV (Figure S9, Supporting Information), indicating its potential in practical application. Figure 3c displays the Tafel plots of the corresponding polarization curves. Remarkably, the Tafel slope of the Co/CoMoN/NF heterostructures electrode was as low as 68.9 mV dec^−1^, which is far lower than the values of 106.1, 120.0, and 106.6 mV dec^−1^ for the CoMoN/NF, CoMoO*_x_*/NF, and Co/NF electrodes, respectively. These results suggest that the Volmer step is significantly accelerated by decorating with Co NPs due to the synergistic effect between Co NPs and CoMoN nanosheets, and the HER kinetics on the Co/CoMoN/NF heterostructures is determined by the Heyrovsky step.^[^ ^22^ ^]^ These results are consistent with the dual‐descriptor design, where Co NPs and CoMoN nanosheet might take charge of hydroxyl and hydrogen adsorption, respectively.^[^ ^21^, ^42^, ^43^ ^]^ In addition, CoMoN possesses higher electric conductivity than CoMoO*_x_*, which is beneficial to charge transfer during the HER process, leading to the enhanced HER activity. Moreover, Co/CoMoN/NF heterostructures have a smaller charge transfer resistance (1.22 Ω) than CoMoN/NF (4.3 Ω), Co/NF (11.6 Ω), and Pt/C/NF (2.1 Ω) electrodes (Figure 3d), indicating enhanced charge transfer property through the Co/CoMoN interface. Electrochemical double layer capacitance (*C*_edl_) was employed to investigate the influence of electrochemical surface areas (ECSAs) on HER activity (Figure S10, Supporting Information). As shown in Figure 3e, the calculated 2*C*_edl_ values of Co/CoMoN/NF, CoMoN/NF, and Co/NF electrodes are 384.2, 246.6, and 3.8 mF cm^−2^, respectively. The larger *C*_edl_ value of Co/CoMoN/NF heterostructures indicates that the Co/CoMoN heterostructures have a higher ECSA, which can provide more active sites for HER. Figure S11, Supporting Information, displays that the current density normalized to 2*C*_edl_ of Co/CoMoN/NF heterostructures (1.57 mA mF^−1^) is 2.3 times higher than that of CoMoN/NF electrode (0.68 mA mF^−1^) at overpotential of 350 mV, whereas the ECSA of Co/CoMoN/NF is only1.5 times higher than that of CoMoN/NF, which indicates that the interface effect rather than the increased ECSA is the key factor for the enhanced HER activity of Co/CoMoN/NF. Moreover, Co/CoMoN/NF heterostructures deliver a 2.25 times higher turn‐over frequency value (0.310 s^−1^) than that of CoMoN/NF (0.138 s^−1^) at the overpotential of 300 mV (Figure S12, Supporting Information), further demonstrating the enhanced intrinsic activity of Co/CoMoN/NF heterostructures.

Since nitridation plays a key role in improving HER activity of Co/CoMoN/NF heterostructures, the influence of nitridation conditions on the HER activity was also investigated. Co/CoMoN/NF heterostructures prepared with different nitridation conditions have similar flower‐like nanosheets microspheres morphology decorated with small Co NPs (Figure S13, Supporting Information). The Co/CoMoN/NF heterostructures prepared at 500 °C for 2 h exhibited the highest HER activity among the prepared electrodes (Figure S14, Supporting Information), indicating that 500 °C for 2 h under NH_3_ atmosphere is the optimum nitridation condition in our work.

The long‐term durability test for HER was conducted at the current density of −100 mA cm^−2^ in 1.0 m KOH to assess the stability of Co/CoMoN/NF heterostructures. As shown in Figure 3f, there is no noticeable increase in the overpotential during 60 h. In addition, after continuous 3000 cyclic voltammetry circles (Figure S15, Supporting Information), the linear sweep voltammetry (LSV) polarization curves overlaid with the initial circle and the morphology (Figure S16, Supporting Information) of Co/CoMoN/NF heterostructures is well maintained. The XRD pattern of Co/CoMoN/NF heterostructures (Figure S17, Supporting Information) after stability test was consistent with that of the fresh catalyst. The high resolution XPS analysis in Figure S18, Supporting Information, indicates that the intensities of Co^0^ and Mo^4+^ peaks increased after stability test, which may be ascribed to the electroreduction of a small amount of Co/Mo oxides/nitrides during the HER. This may be one of the reasons for the slight deactivation of the Co/CoMoN/NF after long‐term durability test. It is worth noting that the HER activity of Co/CoMoN/NF heterostructures is higher than that of most of recently reported non‐noble metal‐based catalysts (Figure 3g and Table S1, Supporting Information) in alkaline media, further suggesting the excellent activity of the Co/CoMoN/NF heterostructures.

**Figure 4:**
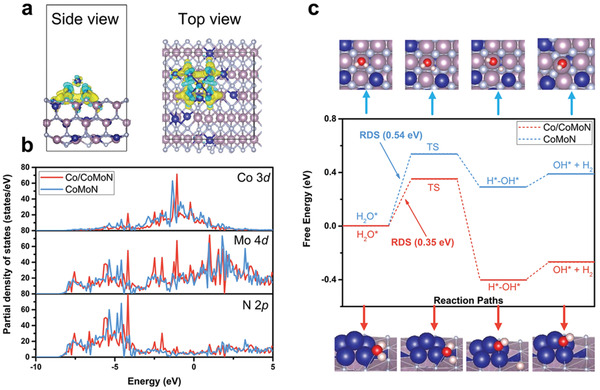
a) Side and top views of charge density difference isosurfaces for Co/CoMoN; light blue and yellow regions indicate electron deficient and accumulation, respectively. b) The partial density of states changes after Co_5_ cluster decorated on the surface of CoMoN. c) Reaction free energy diagram of water dissociation and hydrogen evolution on Co/CoMoN and CoMoN. The corresponding reaction intermediates and transition states are shown below and above the reaction free diagram. Pink, blue, gray, red, and white balls represent Mo, Co, N, O, and H atoms, respectively.

DFT calculations were performed to shed light on the mechanism of excellent HER activity of Co/CoMoN/NF heterostructures. The interfacial structures are shown in Figure S19, Supporting Information, where Co_5_ clusters are fixed on the surface of CoMoN. The electron redistribution after forming the interface structures were described by the charge density difference of Co/CoMoN in **Figure** **4**a. It is found that the Co atoms in Co_5_ cluster are electron deficient and there are lots of electrons accumulated at the interface, which is beneficial to water splitting. The electron distribution is consistent with the Bader charge analysis, where there are 1.18 *e* transferred from Co clusters to CoMoN. As shown in Figure S19, Supporting Information, the Co atoms have positive charge while the adjacent N atoms have negative charge, which are beneficial for the adsorption of hydroxyl and hydrogen, respectively. Moreover, the partial density of states of CoMoN and Co/CoMoN (Figure 4b) demonstrates that Co‐3d, Mo‐4d, and N‐2p orbitals are slightly shifted downwards after forming the interface structures, indicating high surface reactivity. According to the reaction mechanism reported by Prof. Sargent^[^ ^42^ ^]^ and Prof. Zhang,^[^ ^22^ ^]^ Figure 4c displays the reaction free energy of water splitting and hydrogen evolution on CoMoN and Co/CoMoN, and their corresponding reaction intermediates and transition states are shown above and below the reaction free energy plot. Compared with hydrogen evolution, water dissociation is more difficult, which indicates that the rate determining step is water dissociation. Fortunately, the interfacial Co atoms in Co_5_ cluster could provide the active sites for activating water, and therefore, the water dissociation energy barrier is greatly reduced to 0.35 eV from that (0.54 eV) on clean surfaces of CoMoN. The dissociated OH* and H* are adsorbed on Co_5_ cluster and the surface of CoMoN, respectively. And then hydrogen molecule is generated through a satisfactory reaction free energy of 0.12 eV. The calculated catalytic performance on Co/CoMoN is better than those of CrO*_x_*/Cu–Ni^[^ ^42^ ^]^ (0.64 eV) and Ni/NiMoN^[^ ^22^ ^]^ (1.08 eV). As expected, the DFT results are consistent with our experimental results. Based on experimental and theoretical results, the alkaline HER mechanism of the present Co/CoMoN/NF heterostructures can be proposed: Co NPs serve for accelerating the dissociation of water molecule (H_2_O + e^−^ → H* + OH^−^) to generate H*, while CoMoN nanosheets are the active sites for H* adsorption and desorption (2H* → H_2_) to generate H_2_ molecule.

**Figure 5:**
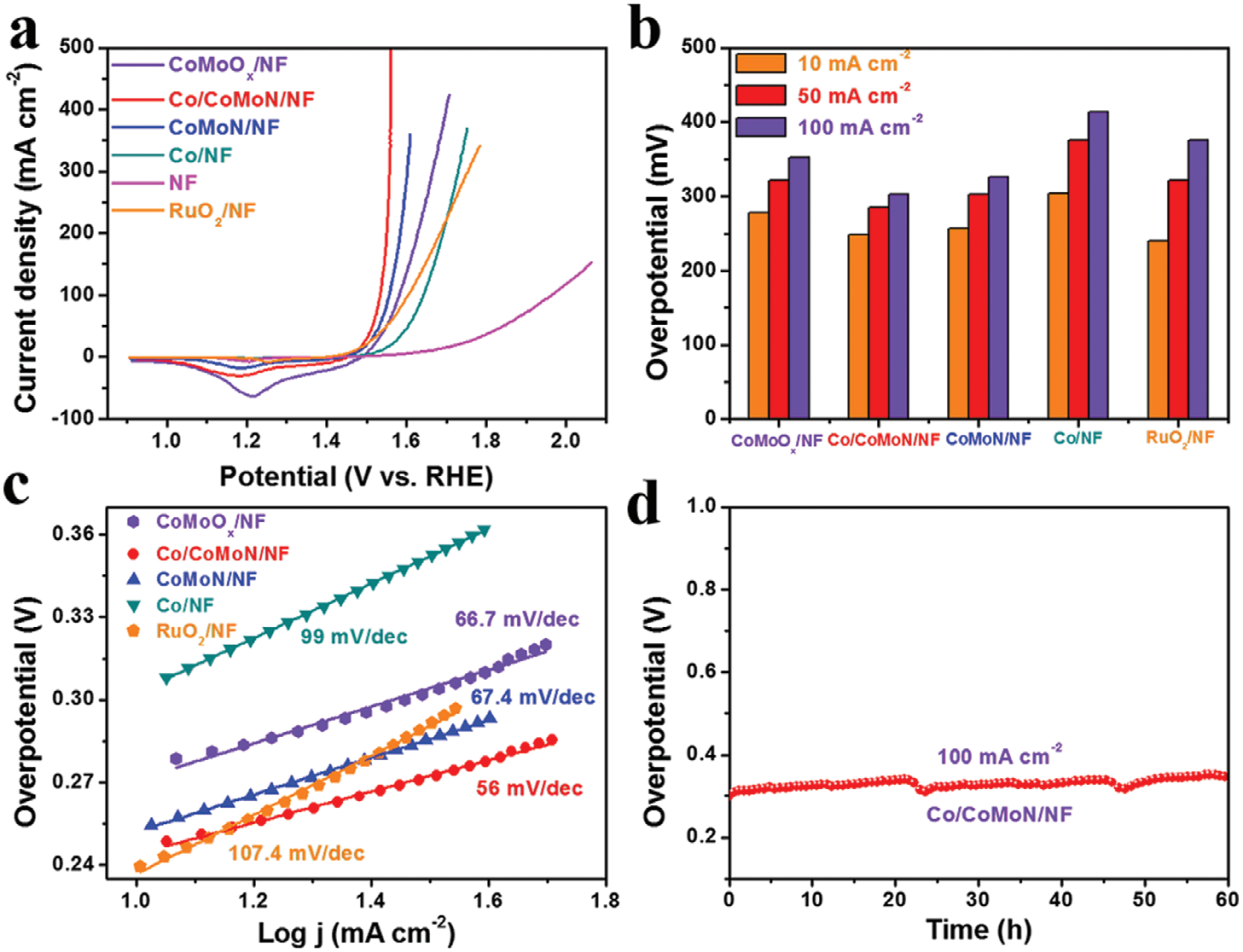
a) Typical polarization curves of CoMoO*_x_*/NF, Co/CoMoN/NF, CoMoN/NF, Co/NF, NF, and RuO_2_/NF, sweep rate: 10 mV s^−1^, electrolyte: 1.0 m KOH. b) Overpotential at current density of 10, 50, and 100 mA cm^–2^ for CoMoO*_x_*/NF, Co/CoMoN/NF, CoMoN/NF, Co/NF, and RuO_2_/NF. c) Tafel plots of different catalysts obtained from the polarization curves in panel (a). d) Stability test of Co/CoMoN/NF at current density of 100 mA cm^−2^ for 60 h in 1.0 m KOH.

Besides the excellent HER activity, Co/CoMoN/NF heterostructures also exhibit outstanding OER activity. For the purpose of eliminating the effect of capacitive current owing to the metal ions oxidation on catalyst performance, LSV curves were measured in the backward direction with a scan rate of 10 mV s^−1^ (**Figure** **5**a). The peaks at around +1.2 V_RHE_ are attributed to the reduction of the oxidized Co species.^[^ ^44^, ^45^ ^]^ The redox peaks of Ni oxides at about +1.4 V_RHE_^[^ ^44^ ^]^ are not obvious probably due to the lower content of Ni species existed in the electrodes. Co/CoMoN/NF heterostructures exhibit the highest OER activity among the as‐prepared catalysts with the smallest overpotential of 303 mV to drive the current density of 100 mA cm^−2^ (Figure 5b), which is lower than that of CoMoN/NF (326 mV), Co/NF (413 mV), and RuO_2_/NF (375 mV) electrodes. The OER kinetics of Co/CoMoN/NF heterostructures was evaluated by Tafel plot, delivering an impressive Tafel slope of 56 mV dec^−1^ (Figure 5c), which is lower than CoMoN/NF (67.4 mV dec^−1^), Co/NF (99 mV dec^−1^), and RuO_2_/NF (107.4 mV dec^−1^) electrodes, manifesting accelerated kinetics on Co/CoMoN interface. Remarkably, the OER performance of Co/CoMoN/NF heterostructures is also competitive compared with recently reported non‐noble metal oxides and hydroxides (Table S2, Supporting Information). The electron transfer from Co NPs to CoMoN nanosheets leads to the formation of positive charge state Co^*δ*+^ on the Co/CoMoN interface, and the underling CoMoN optimized the local electronic structure of Co atoms; the surface electron‐deficient Co sites might be beneficial to OH^−^ adsorption,^[^ ^24^, ^46^, ^47^ ^]^ which could significantly enhance OER activity in alkaline media. The OER durability test was performed at the current density of 100 mA cm^−2^ for 60 h (Figure 5d), showing that there is no noticeable change in the overpotential, indicating its outstanding stability. This is further confirmed by the continuous CV scanning measurement (Figure S20, Supporting Information). In addition, no noticeable structural change was observed after durability test (Figure S21, Supporting Information), further confirming the good stability of the Co/CoMoN/NF heterostructures. After OER test, the XPS spectra (Figure S22, Supporting Information) show that the Co 2p, Mo 3d, and N 1s peaks are shifted slightly to higher binding energies compared to those of the fresh Co/CoMoN/NF, which is ascribed to the small amount of Co, Mo, and N species being oxidized under OER operation potentials. However, the XRD measurements (Figure S23, Supporting Information) show that the crystal structure of Co/CoMoN/NF is maintained well after OER test. The above results indicate that such an oxidation process only occurs on the surface of Co/CoMoN/NF, and thus the morphology and crystal structure are maintained well.

The reaction mechanism of OER on Co/CoMoN/NF heterostructures was further demonstrated by DFT calculations. Based on experimental results, Co atoms will be partially oxidized during OER process; therefore, we built partially oxidized Co clusters supported on CoMoN as the active center for OER, as shown in Figure S24, Supporting Information. Following H_2_O dissociation, hydroxyl is adsorbed on Co atoms. The next three steps are as follows: OH* + OH^−^ → O* + H_2_O + e^−^; O* + OH^−^ → OOH* + e^−^; OOH* + OH^–^ → O_2_* + H_2_O + e^−^, as shown in Figure S25, Supporting Information. Their reaction free energy values are 1.04, 1.85, and 1.06 eV, respectively. It means that the potential limiting step is O* + OH^−^ → OOH* + e^−^ with the small overpotential of 0.62 V, which is consistent with the outstanding catalytic performance for OER in the experiment.

To study the influence of composition on the catalytic activity, Co/CoMoN/NF heterostructures with different Co/Mo ratios were prepared for HER and OER by tuning the mole ratio of metal precursors. It was found that the Co/CoMoN/NF heterostructures prepared with Co(NO_3_)_2_∙6H_2_O/(NH_4_)_6_Mo_7_O_24_·4H_2_O mole ratio of 5/2 exhibits the highest HER and OER activities (Figure S26, Supporting Information). In addition, Co/CoMoN/CC catalyst shows much lower HER and OER activities than that of Co/CoMoN/NF (Figure S27, Supporting Information), indicating that the use of NF as a substrate is beneficial for enhancing catalytic activity due to its high electric conductivity and unique porous structure. To investigate the effect of the small amount of Mo oxides such as MoO_2_ and MoO_3_ on the electrocatalytic activity, the MoN/NF was synthesized for HER and OER by the same procedure as Co/CoMoN/NF except that the Co(NO_3_)_2_∙6H_2_O was not used as metal precursor. The results show that MoN/NF exhibits inferior HER and OER activities than Co/CoMoN/NF (Figure S28, Supporting Information), which indicates that the small amount of Mo oxides are not the main factors for the high performance of Co/CoMoN/NF. Moreover, replacing (NH_4_)_6_Mo_7_O_24_·4H_2_O by Ni(NO_3_)_2_·6H_2_O and Fe(NO_3_)_3_·9H_2_O, respectively, the obtained Co/CoNiN/NF and Co/CoFeN/NF catalysts also exhibit much lower HER and OER activities than that of Co/CoMoN/NF (Figure S29, Supporting Information), suggesting the combination of Co and Mo is the optimal choice for alkaline HER/OER.

**Figure 6:**
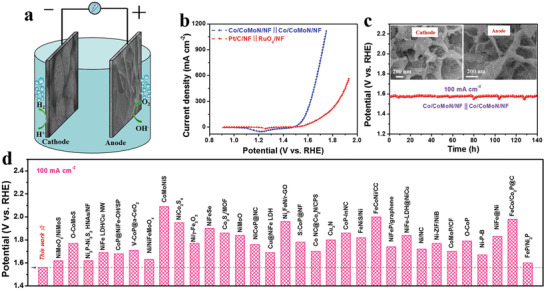
a) Schematic illustration of water splitting powered by electronic energy, where alkaline electrolyzer is constructed with Co/CoMoN/NF both as cathode and anode. b) Representative polarization curves for overall water splitting in cells of Co/CoMoN/NF||Co/CoMoN/NF and Pt/C/NF||RuO_2_/NF, within 1.0 m KOH electrolyte, sweep rate: 10 mV s^−1^. c) Electrochemical stability test of Co/CoMoN/NF||Co/CoMoN/NF cell at 100 mA cm^−2^ for 140 h. d) Comparison of working voltage of Co/CoMoN/NF‐based electrolyzer at the current density of 100 mA cm^−2^ with the values of ones reported previously.

Encouraged by the extraordinary activity of Co/CoMoN/NF heterostructures for HER and OER in alkaline media, an alkaline electrolyzer is assembled with Co/CoMoN/NF serving as both the cathode and anode, as illustrated in **Figure** **6**a. It delivers small voltages of 1.50 and 1.56 V to reach the current densities of 10 and 100 mA cm^−2^ (Figure 6b), respectively, for overall water splitting. This performance far beyond outperforms commercially available Pt/C/NF||RuO_2_/NF system, which requires cell voltages of 1.55 and 1.70 V to reach the same current densities. Meanwhile, the Co/CoMoN/NF system also exhibited excellent stability over 140 h at the current density of 100 mA cm^−2^ for full water splitting (Figure 6c), which is further confirmed by the retaining morphology (Figure 6c inset) after durability test and the continuous CV scanning measurement (Figure S30, Supporting Information). A small amount of flocculent structures were observed at the anode probably due to the surface oxidation of Co/CoMoN/NF heterostructures during the continuous operating for 140 h at high current density. Remarkably, this overall water splitting performance outperforms most of recently reported nonprecious metal‐based catalysts (Figure 6d and Table S3, Supporting Information), implying distinguished candidate for concurrent H_2_ production.

## 3 Conclusions

In summary, charge polarized Co/CoMoN/NF heterostructures were designed and constructed for water splitting in alkaline media. Co/CoMoN/NF heterostructures can deliver current density of 100 mA cm^−2^ at overpotentials of 173 mV and Tafel slope of 68.9 mV dec^−1^. For OER, Co/CoMoN/NF heterostructures deliver the current density of 100 mA cm^−2^ at overpotential of 303 mV with a Tafel slope of 56 mV dec^−1^. What's more, when assembled as an alkaline electrolyzer, Co/CoMoN/NF system exhibits an extremely low voltage of 1.56 V to reach the current density of 100 mA cm^−2^ for full water splitting along with superior long‐term durability. This study proposes a promising strategy toward efficient water splitting catalyst design. The novel interfacial design strategy enlightens the construction of promising heterostructures for efficient water splitting and beyond.

## Conflict of Interest

The authors declare no conflict of interest.

## Supporting information

Supporting Information

## Data Availability

The data that support the findings of this study are available from the corresponding author upon reasonable request.
