# Supplementary material for: Interface Engineering of Co/CoMoN/NF Heterostructures for High‐Performance Electrochemical Overall Water Splitting
Source: Adv Sci (Weinh). 2022 Feb 11;9(11):2105313. doi: 10.1002/advs.202105313 (PMC9009127; doi:10.1002/advs.202105313)
Supplement: Supplementary file 1 — Supporting Information [file ADVS-9-2105313-s001.pdf]

ADVANCED SCIENCE Open Access

Supporting Information

for *Adv. Sci.*, DOI: 10.1002/advs.202105313

Interface engineering of Co/CoMoN/NF heterostructures for high-performance electrochemical overall water splitting

Haibin Ma,‡, Zhiwen Chen,‡ Zhili Wang, Chandra Veer Singh*, and Qing Jiang**

Supporting Information

Interface engineering of Co/CoMoN/NF heterostructures for high-performance electrochemical overall water splitting

Haibin Ma,[‡,a] Zhiwen Chen,[‡,b] Zhili Wang[a], Chandra Veer Singh[*b,c], and Qing Jiang*[a]*

Haibin Ma,[‡,a] Zhili Wang*[a], Qing Jiang*[a]

[a] Key Laboratory of Automobile Materials, Ministry of Education, and School of Materials Science and Engineering, Jilin University, Changchun 130022, China.

[*]E-mail: zhiliwang@jlu.edu.cn; Jiangq@jlu.edu.cn

Zhiwen Chen,[‡,b] Chandra Veer Singh[*b,c]

[b] Department of Materials Science and Engineering, University of Toronto, 184 College Street, Suite 140, Toronto, ON M5S 3E4, Canada.

Chandra Veer Singh[*b,c]

[c] Department of Mechanical and Industrial Engineering, University of Toronto, 5 King's College Road, Toronto, ON M5S 3G8, Canada.

[*]E-mail: chandraveer.singh@utoronto.ca

‡These authors contributed equally to this work.

Experimental Section

1. Chemicals

Potassium hydroxide (KOH), cobalt nitrate hexahydrate ($Co(NO_3)_2 \cdot 6H_2O$), ammonium fluoride (NH_4F), and urea ($CO(NH_2)_2$) were purchased from Sinopharm Chemical Reagents Co., Ltd. (SCRC, China). All the chemicals were used as received without further purification. The nickel foam (NF) was purchased from Sheng Qiang Co., Ltd. (Jiangsu, China). Prior to use, it was treated by means of degreasing treatments to remove surface oxide layers.

2. Materials synthesis

2.1. Synthesis of $CoMoO_x$/NF

A piece of NF was degreased in acetone solution and etched in 3.0 M HCl solution with sonication for 30 min, subsequently washed with deionized water and ethanol to ensure a clean surface. $CoMoO_x$ was synthesized through a simple hydrothermal reaction. In a typical synthesis, 2.5 mmol urea, 2.0 mmol NH_4F, 0.25 mmol $Co(NO_3)_2 \cdot 6H_2O$, and 0.1 mmol $(NH_4)_6Mo_7O_{24} \cdot 4H_2O$ were dissolved in 40 mL deionized water to form a clear solution under constant stirring. Then the pre-treated NF was transferred into a Teflon-lined autoclave (100 mL) containing the above solution and maintained at 120 ^oC for 12 h. After natural cooling to room temperature, $CoMoO_x$ was taken out, washed by deionized water and ethanol, finally dried at 60 ^oC under vacuum for 24 h, the obtained sample is termed as $CoMoO_x$/NF.

2.2. Synthesis of Co/CoMoN/NF heterostructures

Co/CoMoN/NF heterostructures were obtained by annealing $CoMoO_x$/NF at 500 ^oC for 2h under NH_3 atmosphere.

2.3. Synthesis of Co/CoMoN/CC heterostructures

Co/CoMoN/CC heterostructures were obtained with the same procedure as Co/CoMoN/NF heterostructures except utilizing carbon cloth substitute for Ni foam in the hydrothermal reaction.

2.4. Synthesis of CoMoN/NF

CoMoN/NF was obtained by acid etching of Co/CoMoN/NF heterostructures in 0.1 M HCl for 90 min.

2.5. Synthesis of Co/NF

Co/NF was prepared by the same procedure with Co/CoMoN/NF heterostructures except without introduction of Mo source in the hydrothermal reaction and annealing under H_2 atmosphere.

2.6. Synthesis of Pt/C/NF electrode

Pt/C catalyst inks were prepared by mixing commercial-available Pt/C (20 wt%) in a Nafion (0.05 wt%) solution containing isopropanol (20%) and water (80%) under rigorous sonication for 30 min to ensure homogeneous suspension. Total 400 μL Pt/C inks were drop-cast onto NF (10 mm × 20 mm) to prepare Pt/C/NF electrode for

electrochemical measurements.

2.7. Synthesis of RuO_2/NF electrode

RuO_2 catalyst inks were prepared by mixing commercial-available RuO_2 in a Nafion (0.05 wt%) solution containing isopropanol (20%) and water (80%) under rigorous sonication for 30 min to ensure homogeneous suspension. Total 400 μL RuO_2 inks were drop-cast onto NF (10 mm × 20 mm) to prepare RuO_2/NF electrode for electrochemical measurements.

3. Electrochemical measurements

The HER and OER tests were performed in a three-electrode system using a platinum plate and normal calomel as the counter electrode and reference electrode, respectively. Prior to recording the polarization curves, all the samples were activated by the cyclic voltammetry (CV) test at 10 mV s^{-1}. The electrochemical impedance spectroscopy (EIS) was conducted at 280 mV overpotential for both HER and OER from 100 kHz to 0.01 Hz with 10 mV alternate current (AC) amplitude. According to the previous reports,[1-2] all the electrochemical data for OER and HER were obtained with 85% iR-compensation in our work. Faradaic efficiency for HER was obtained by a gas-chromatograph (Shimadzu GC-2014). The long-term stability performance for HER, OER and full water electrolysis was operated at the current densities of 100 mA cm^{-2}. O_2- and Ar -saturated 1.0 M KOH solutions were used as the electrolytes for OER and HER tests, respectively, by continuously feeding O_2 and Ar gases, respectively. The rapid cycling stability of Co/CoMoN/NF heterostructures for HER,

OER and full water splitting was tested at 100 mV s^{-1} for 3000 CV cycles and the linear sweep voltammetry (LSV) polarization curves after cycling were recorded for comparison. The electrochemical active surface areas (ECSAs) were assessed from the electrochemical double layer capacitance (C_{edl}), which was calculated by measuring the non-Faradaic capacitive from a series of CV curves at different scan rates (5, 10, 15, 20, 25 mV s^{-1}). By plotting the Δj (j_a-j_c) at 0.15 V vs. RHE for HER against the scan rates, the slope of fitting lines which is twice of the C_{edl} value was used to represent the ECSAs.

4. Calculation of Faradaic efficiency

For Faradaic efficiency measurements, constant potential tests were conducted at the overpotentials of 400, 500, and 600 mV, respectively. After controlling potential electrolysis for 30 min to obtain a steady state, the gas-phase product was analyzed by online gas chromatograph (Shimadzu GC-2014) with a thermal conductivity detector for detecting H_2. The corresponding Faradaic efficiencies were calculated on the basis of the following equation:[3]

$$\mathrm{FE\%} = \frac{\mathrm{v} \times 10^{-6} \times \mathrm{V_{Ar}} \times 10^{-6} \times 96485.3 \times \alpha \times 101300}{8.314 \times 298.15 \times \mathrm{i} \times 60} \times 100\%$$

Where V_{Ar} is the flow rate of carrier gas Ar (20 mL min^{-1}), v (ppm) is the H_2 concentration taking up of the whole gas content, α is the quantity of transferred electron for producing H_2, and i (A) is the test current at a steady state.

5. Calculation of turn-over frequency (TOF)

The TOF values for as-prepared catalysts were calculated by the following equation:[4]

$$\mathrm{TOF} = \frac{\#\text{total hydrogen turn overs/geometric area (cm}^2)}{\#\text{active sites/geometric area (cm}^2)}$$

The total number of hydrogen turnovers were calculated from the current densities at overpotential of 300 mV according to:

$$\#\mathrm{H_2} = (\mathrm{j}\frac{\mathrm{mA}}{\mathrm{cm^2}})(\frac{1\mathrm{C\ s^{-1}}}{1000\mathrm{mA}})(\frac{1\mathrm{mol\ e^{-1}}}{96485.3\ \mathrm{C}})(\frac{1\mathrm{mol\ H_2}}{2\ \mathrm{mol\ e^{-1}}})(\frac{6.022\times10^{23}\mathrm{H_2\ molecules}}{1\ \mathrm{mol\ H_2}})$$

The active sites (including Co and Mo) per real surface area were calculated from the following formula:

$$\#\text{active sites} = (\frac{\text{number of atoms/unit cell}}{\text{Volume/unit cell}})^{2/3}$$

The real surface area for HER is calculated from the electrochemical surface area (ECSA), which can be converted from the specific capacitance (C_s). The ECSAs for each catalyst are obtained from the formula: $A_{ECSAs} = C_{edl}/C_s$. The C_s for ECSAs calculation is 60 μF cm^{-2}.

6. Materials characterization

The morphology and structure of samples was characterized by a scanning electron microscopy (SEM, JSM-6700F, 5k eV). Low-magnification and high-resolution transition electron microscopy (TEM) images were obtained from a TEM (JEOL JEM-2100F, 200k eV) equipped with an energy diffraction spectroscopy (EDS). X-ray diffraction (XRD) measurements were conducted on a D/max 2500pc diffractometer with a mono-chromated Cu Kα radiation (λ=1.54178 Å). X-ray photoelectron spectroscopy (XPS) was performed on a thermal ECSALAB 250 (15k

eV, 6 mA) with an Al anode. All the charge states were compensated by shifting binding energies based on the C 1s peak (284.8 eV). The concentration of metal ions in electrodes was determined by the inductively coupled plasma optical emission spectrometer (ICP-OES, Varian 710-ES).

7. DFT calculations

The Vienna Ab initio Simulation Package (VASP) was applied to perform spin-polarized density functional theory (DFT) calculations.[5] The interaction between valence electrons and ionic cores was described by the Projector augmented wave (PAW) pseudopotential and Perdew-Burke-Ernzerhof (PBE) functional of the generalized gradient approximation (GGA) was considered for the exchange-correlation effects.[6-7] The wave function calculations used the kinetic energy cutoff of 550 eV with the smearing width of 0.03 eV. The vacuum gap was set to more than 15 Å, which was enough for avoiding the interactions between the systems and its mirror images. The van der Waals interaction was considered by Zero damping DFT-D3 method of Grimme.[8] During the geometry optimization process, the Monkhorst-Pack scheme with the k-point was set to be (2, 2, 1) and the convergence criteria for energy and force were 10^{-5} eV and 0.02 eV/Å, respectively. The electron transfer between Co_5 cluster and CoMoN was calculated by Bader charge analysis.[9] In order to calculate the transition state for investigating the energy barrier of water dissociation, the climbing image nudged elastic band (CINEB) method was used.[10]

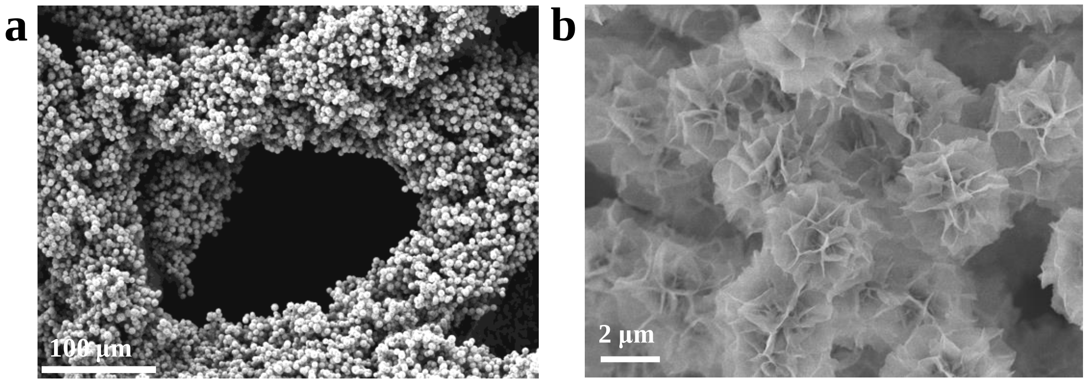

Figure S1. (a) Low magnification and (b) high magnification SEM images of $CoMoO_x$/NF.

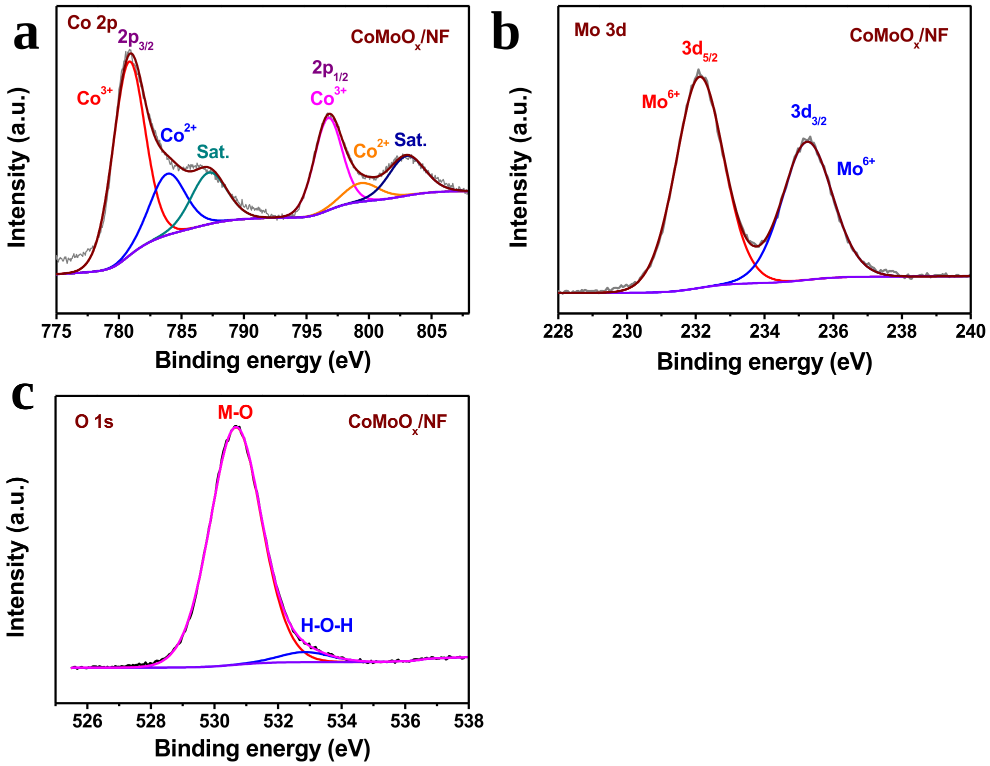

Figure S2. High-resolution XPS spectra of (a) Co 2p, (b) Mo 3d, and (c) O 1s for $CoMoO_x$/NF.

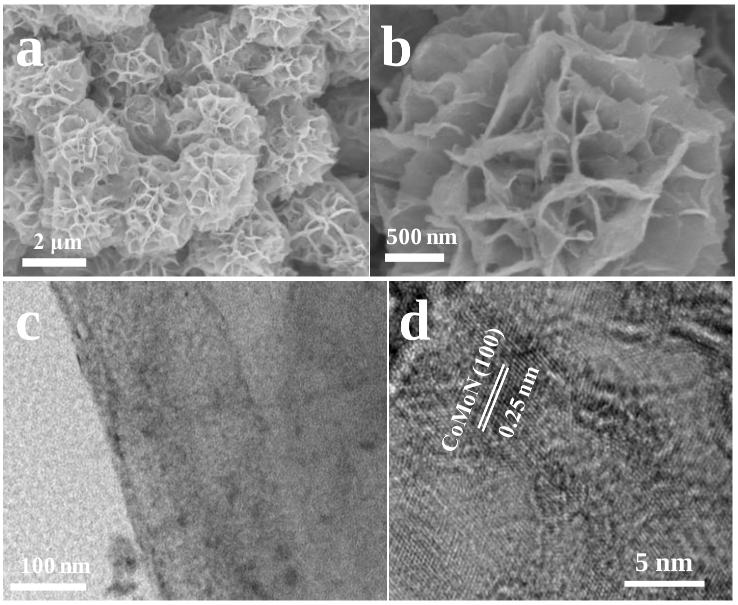

Figure S3. SEM images of (a, b) CoMoN/NF, and (c, d) TEM and HRTEM images of CoMoN/NF.

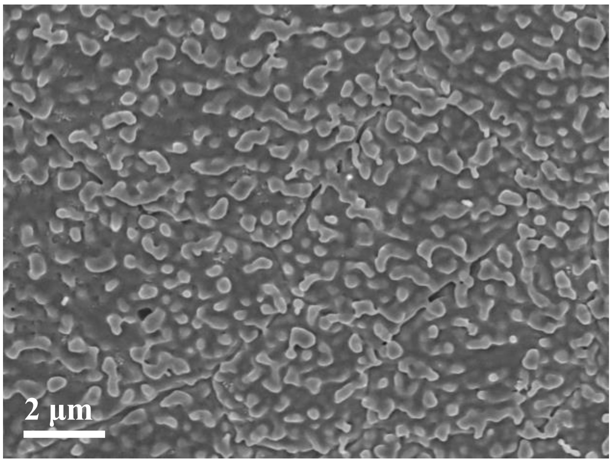

Figure S4. SEM image of Co/NF.

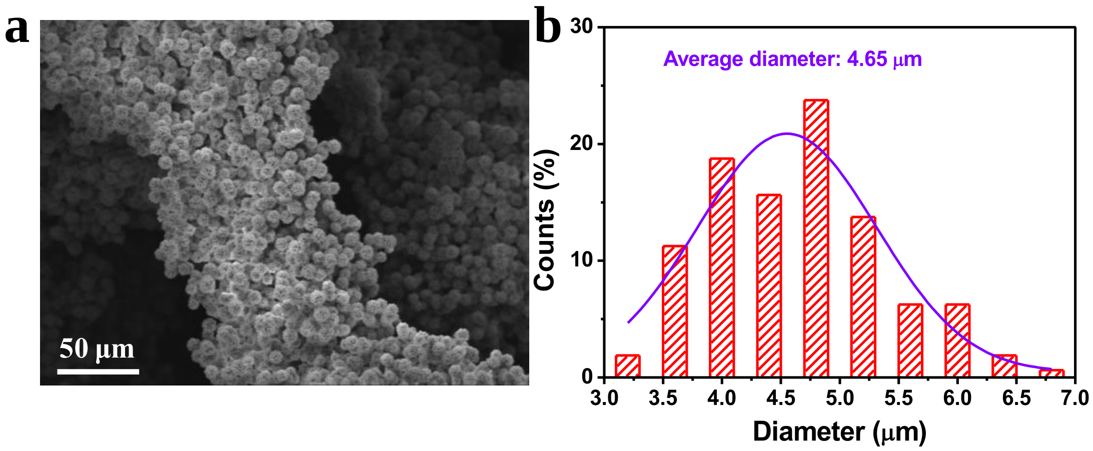

Figure S5. (a) Low-magnification image of Co/CoMoN/NF and (b) diameter distribution of Co/CoMoN microspheres.

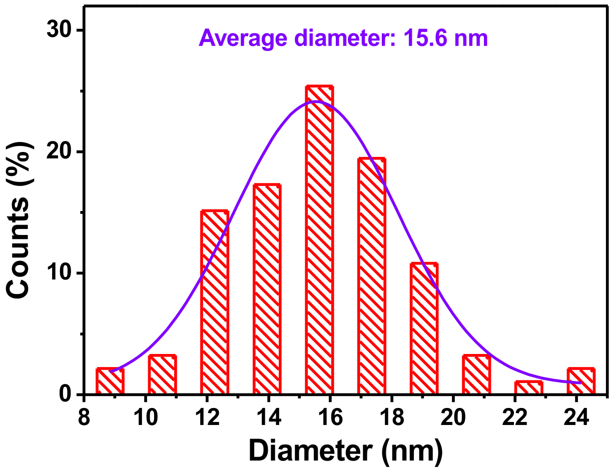

Figure S6. The diameter distribution of Co nanoparticles on Co/CoMoN/NF.

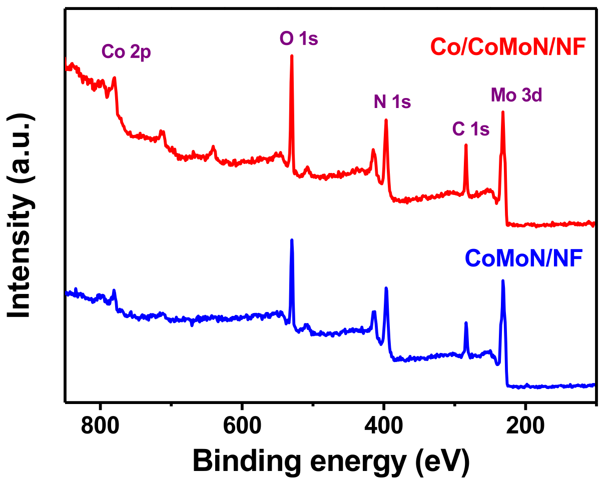

Figure S7. XPS survey spectra of Co/CoMoN/NF and CoMoN/NF.

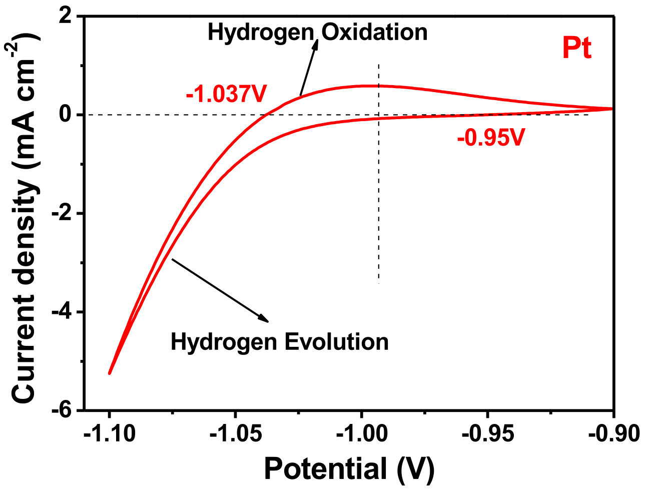

Figure S8. Calibration of calomel normal reference electrode.

Cyclic voltammetry curve of Pt plate as working electrode was performed in H_2-saturated 1.0 M KOH electrolyte. Sweep rate: 1.0 mV/s. The average value (0.994 V) of two potentials at which the current crossed zero was used as the thermodynamic potential for the hydrogen electrode reaction. In H_2-saturated 1.0 M KOH electrolyte, $E_{(\mathrm{RHE})} = E_{(\mathrm{Calomel\ normal\ electrode})} + 0.994\mathrm{V}$.

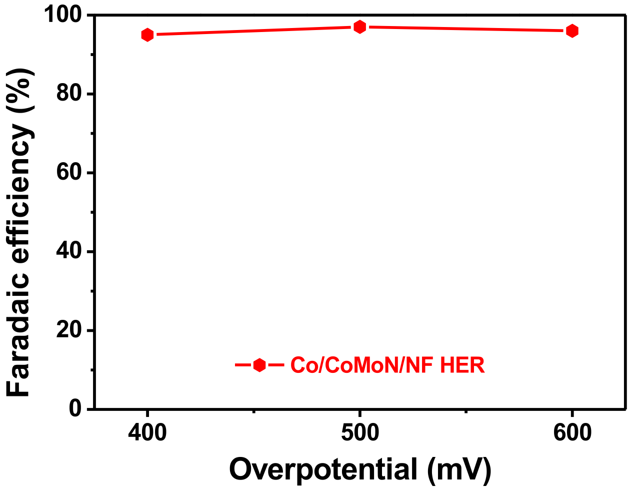

Figure S9. Faradaic efficiencies of Co/CoMoN/NF for HER at overpotentials of 400, 500, and 600 mV.

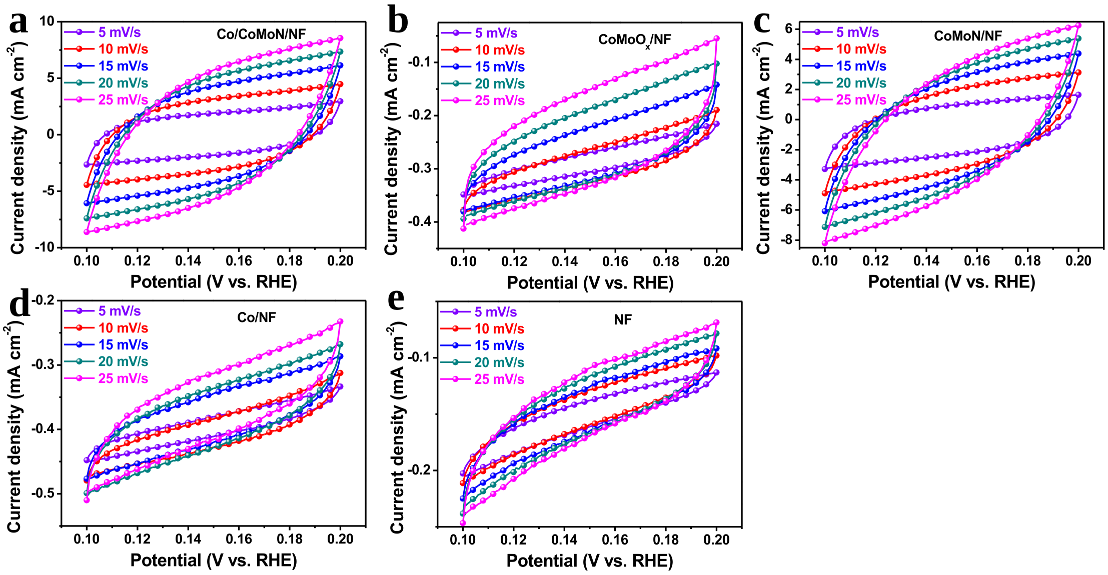

Figure S10. Cyclic voltammograms in the potential window from 0.1 V to 0.2 V (vs. RHE) for (a) Co/CoMoN/NF, (b) $CoMoO_x$/NF, (c) CoMoN/NF, (d) Co/NF, and (e) NF at the scan rates of 5, 10, 15, 20, 25 mV/s.

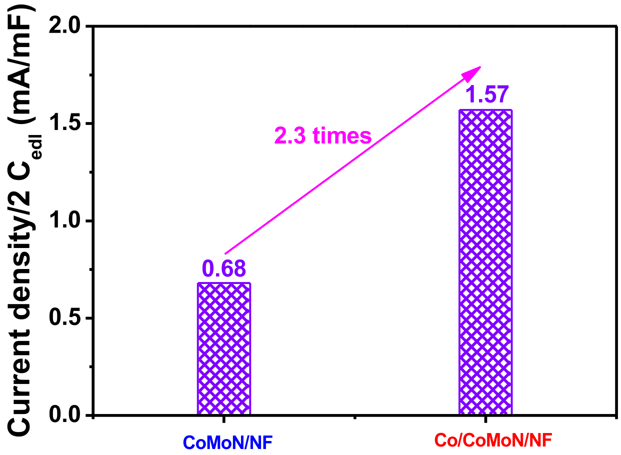

Figure S11. The current densities normalized to $2C_{edl}$ of Co/CoMoN/NF and CoMoN/NF for HER at overpotential of 350 mV.

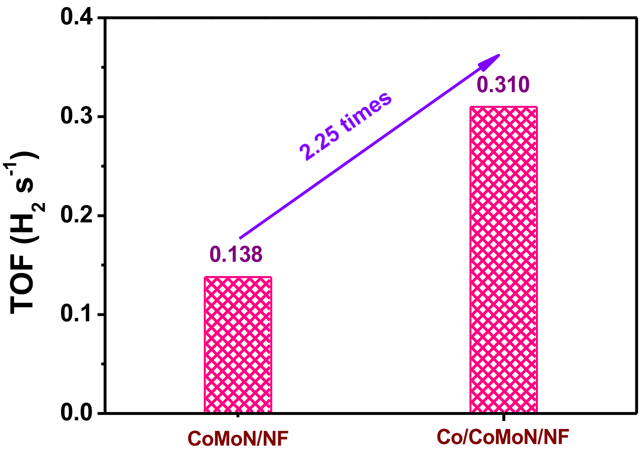

Figure S12. The calculated turn-over frequency (TOF) numbers of Co/CoMoN/NF and CoMoN/NF for HER at overpotential of 300 mV.

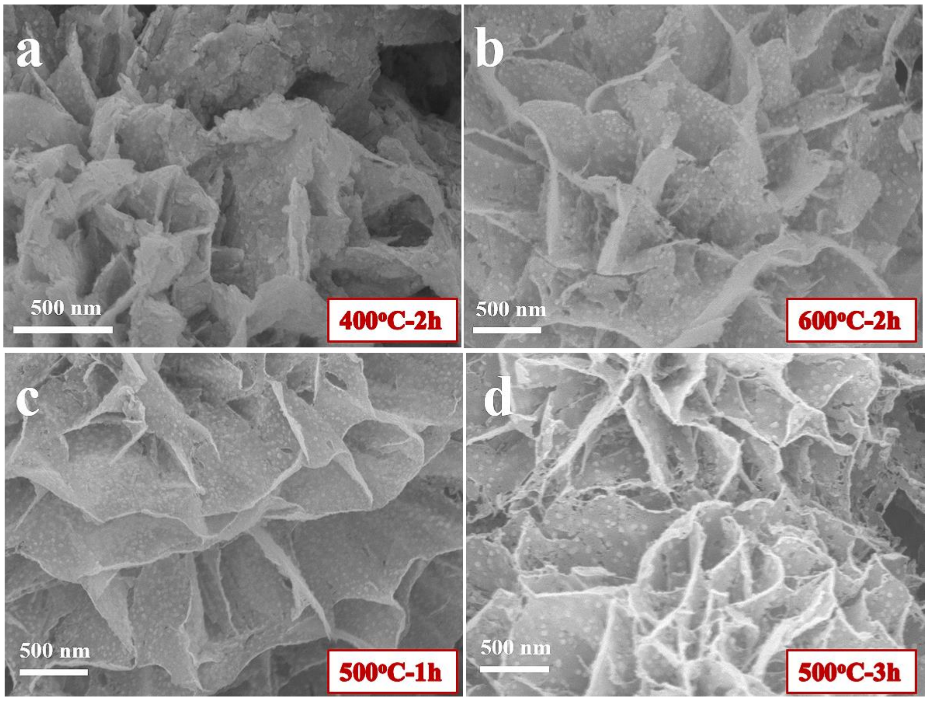

Figure S13. SEM images of Co/CoMoN/NF annealed at (a) 400 ^{o}C for 2h, (b) 600 ^{o}C for 2h, (c) 500 ^{o}C for 1h, and (d) 500 ^{o}C for 3h.

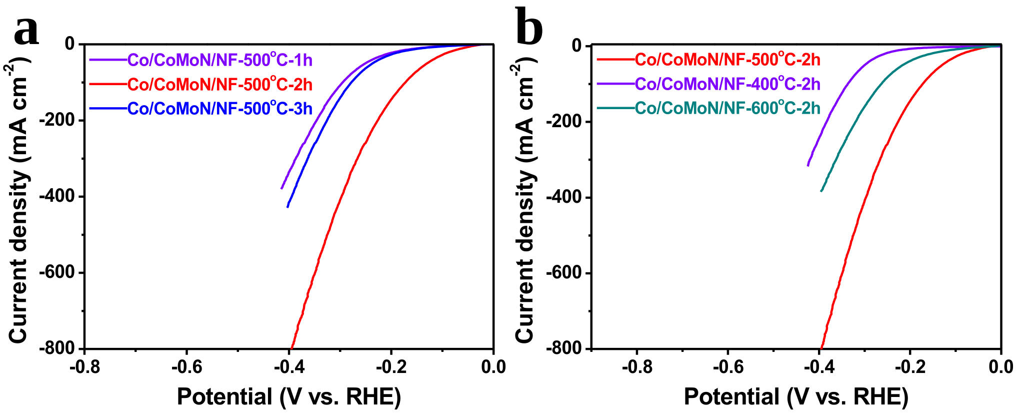

Figure S14. (a) Polarization curves of Co/CoMoN/NF annealed at 500 °C for 1 h, 2 h and 3 h and (b) at 400°C, 500°C and 600°C for 2 h. Sweep rate: 10 mV/s, electrolyte: 1.0 M KOH.

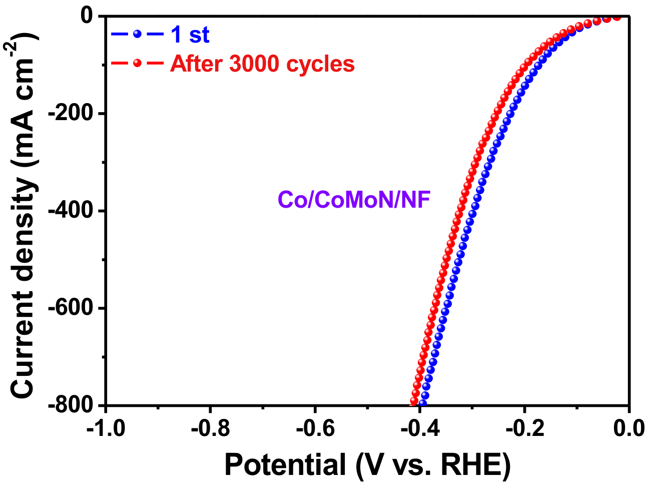

Figure S15. Polarization curves recorded from Co/CoMoN/NF heterostructures for HER at a scan rate of 10 mV/s before (blue curve) and after (red curve) the 3000 cycles.

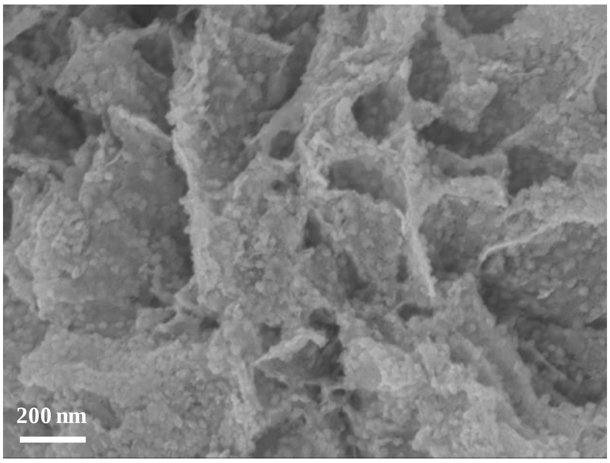

Figure S16. SEM image of Co/CoMoN/NF after HER stability test.

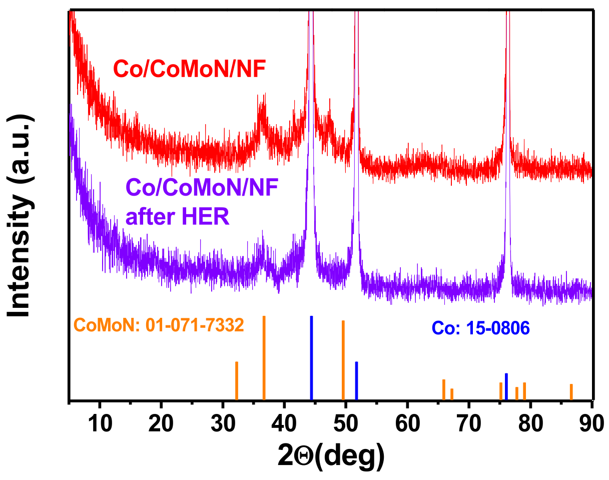

Figure S17. XRD patterns of Co/CoMoN/NF before and after HER stability test.

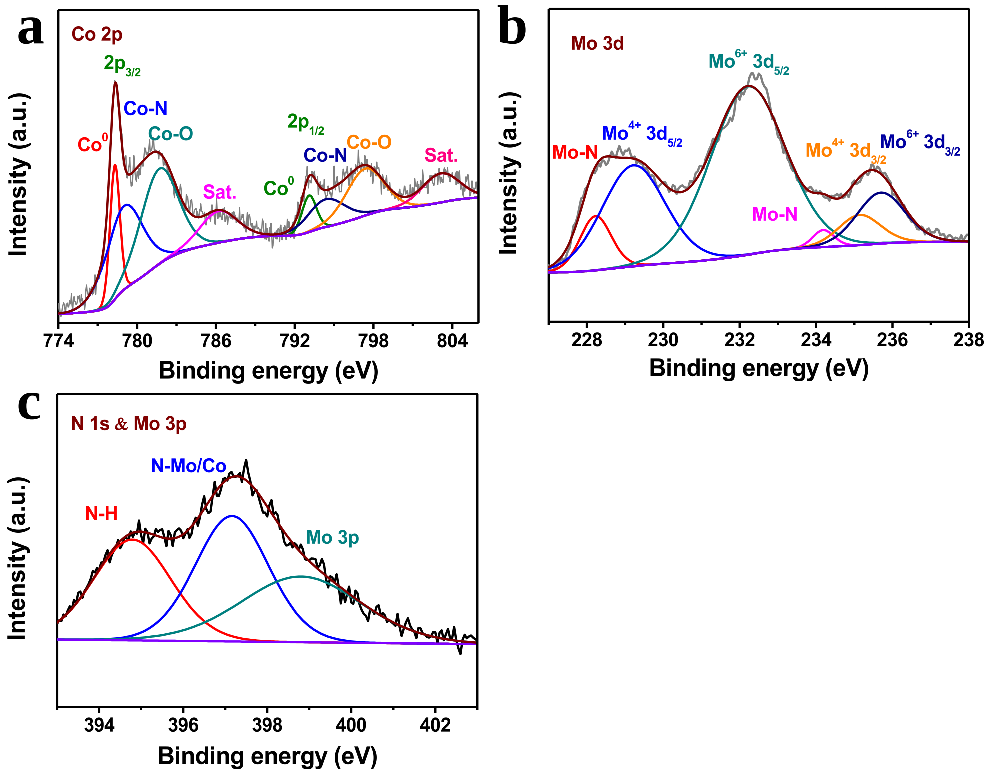

Figure S18. High-resolution XPS spectra of (a) Co 2p, (b) Mo 3d, (c) N 1s and Mo 3p for Co/CoMoN/NF after HER stability test.

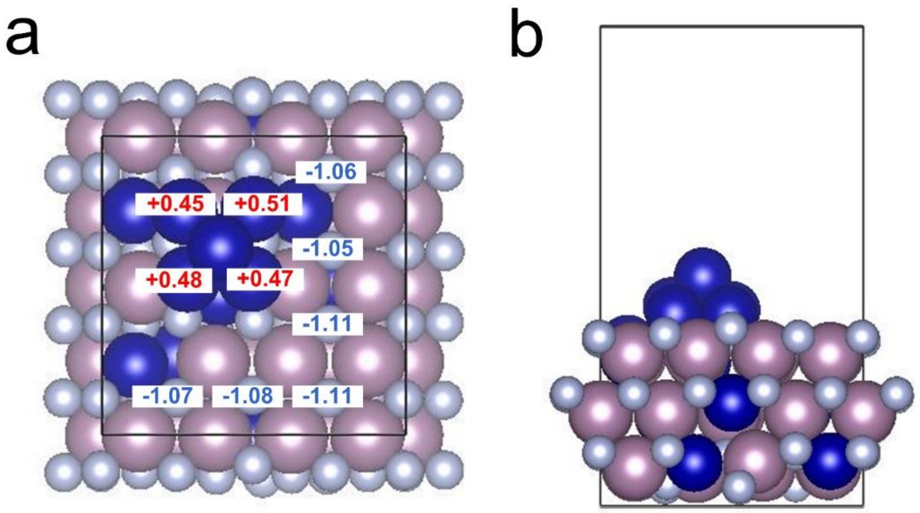

Figure S19. Top (a) and side (b) views of Co_5/CoMoN structure. Pink, blue, and gray balls represent Mo, Co, and N atoms, respectively. The values in (a) indicate the charge distribution of the corresponding atom.

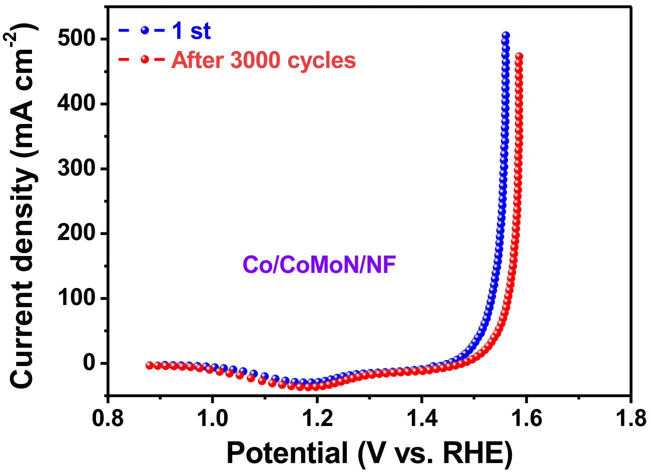

Figure S20. Polarization curves recorded from Co/CoMoN/NF heterostructure for OER at a scan rate of 10 mV/s before (blue curve) and after (red curve) the 3000 CV curves.

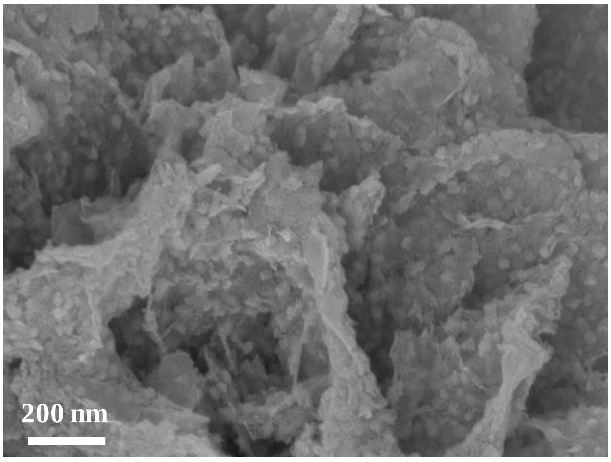

Figure S21. SEM image of Co/CoMoN/NF after OER stability test.

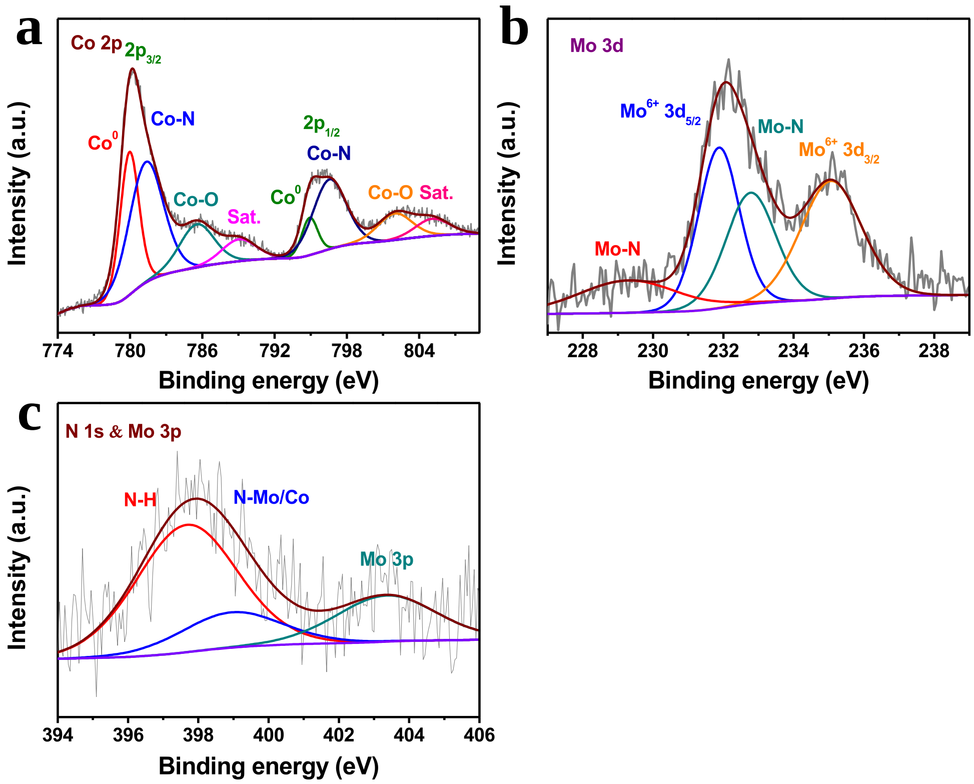

Figure S22. High-resolution XPS spectra of (a) Co 2p, (b) Mo 3d, (c) N 1s and Mo 3p for Co/CoMoN/NF after OER test.

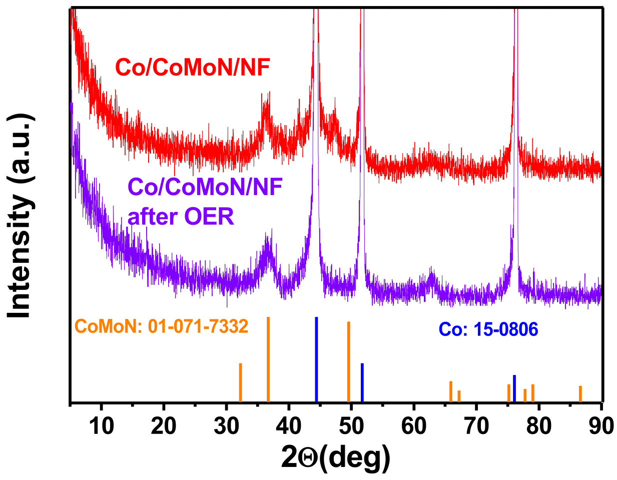

Figure S23. XRD patterns of Co/CoMoN/NF before and after OER stability test.

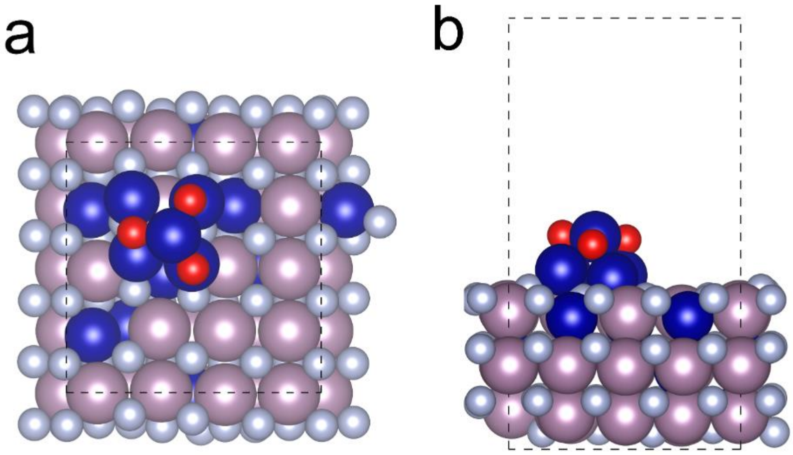

Figure S24. Top (a) and side (b) views of partially oxidized Co_5/CoMoN structure. Pink, blue, gray, red balls represent Mo, Co, N, and O atoms, respectively.

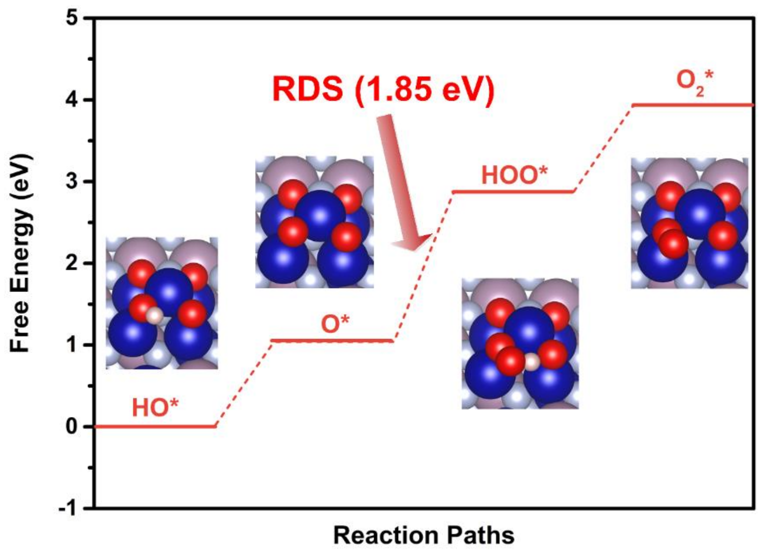

Figure S25. Reaction free energy diagram of OER on partially oxidized Co/CoMoN, including the corresponding reaction intermediates. Pink, blue, gray, red, and white balls represent Mo, Co, N, O, and H atoms, respectively.

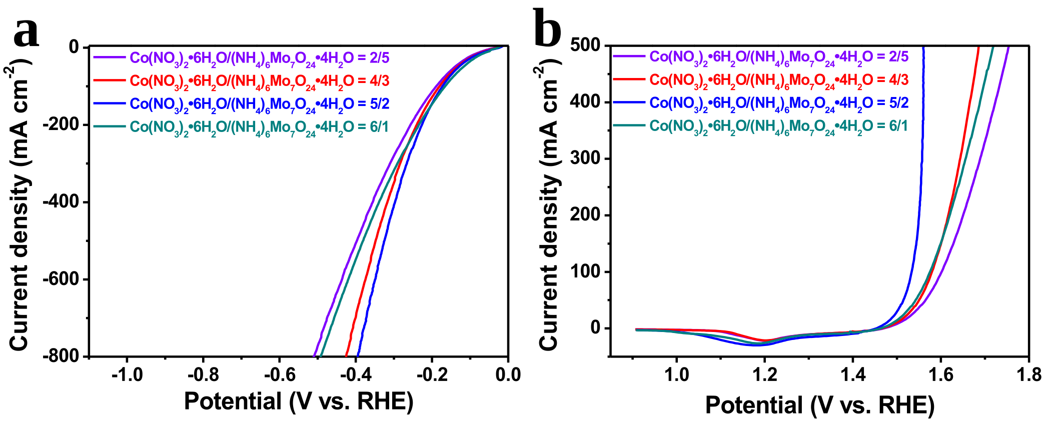

Figure S26. The HER and OER performances of Co/CoMoN/NF heterostructures prepared with different mole ratios of metal precursors. (10 mV/s in 1.0 M KOH.)

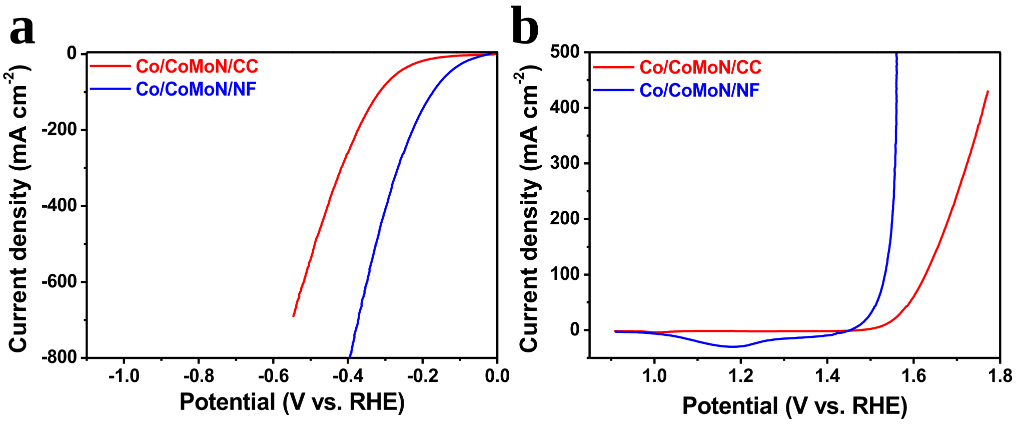

Figure S27. Polarization curves of Co/CoMoN/NF and Co/CoMoN/CC catalysts for (a) HER and (b) OER at a scan rate of 10 mV/s in 1.0 M KOH.

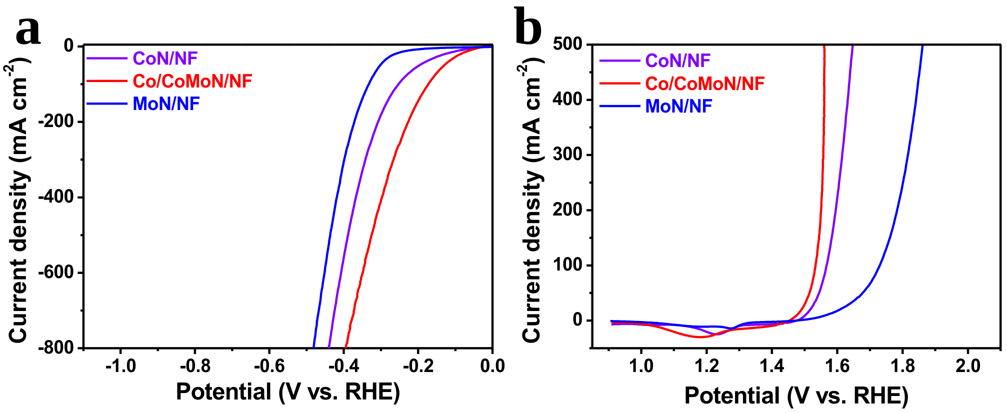

Figure S28. Polarization curves of CoN/NF, MoN/NF and Co/CoMoN/NF catalysts for (a) HER and (b) OER at a scan rate of 10 mV/s in 1.0 M KOH.

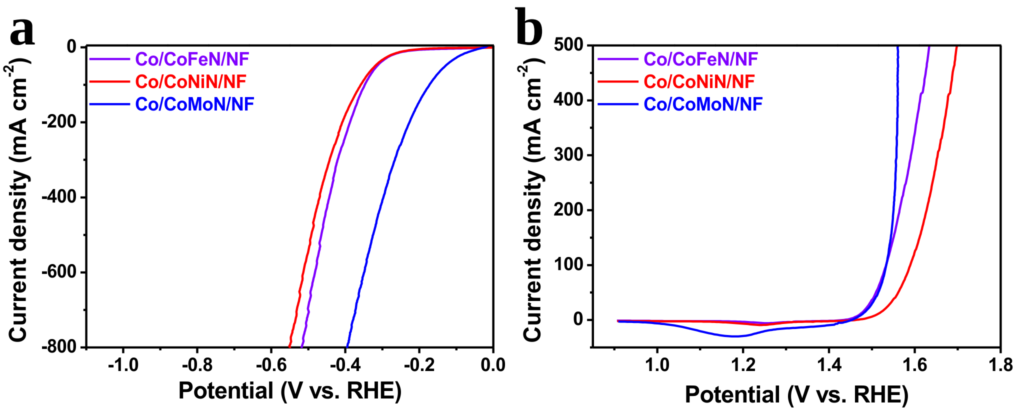

Figure S29. Polarization curves of Co/CoMoN/NF, Co/CoNiN/NF and Co/CoFeN/NF heterostructures for (a) HER and (b) OER at a scan rate of 10 mV/s in 1.0 M KOH.

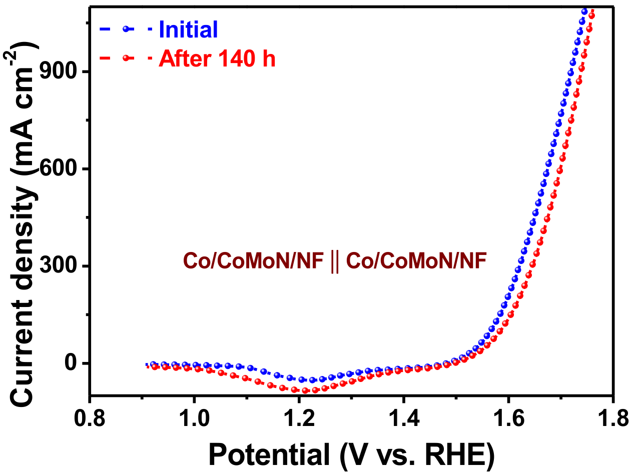

Figure S30. Polarization curves recorded from Co/CoMoN/NF heterostructures for overall water splitting at a scan rate of 10 mV/s before (blue curve) and after (red curve) the chronopotentiometry test at 100 mA/cm^2 for 140 h.

Table S1: Comparison of the HER activity of Co/CoMoN/NF with other non-noble metal based electrocatalysts in alkaline electrolytes.

Catalysts	Electrolyte	Overpotential at -10 mA cm^{-2} (mV)	Tafel slope (mV/dec)	Reference
Co/CoMoN/NF	1.0 M KOH	61	68.9	*This work*
$Ni/Ni(OH)_2$	1.0 M KOH	77	53	[11]
Ni-Mo-$Cu_{0.06}$	6.0 M KOH	86	42	[12]
CoFeO@BP	1.0 M KOH	88	51	[13]
N-NiMoS	1.0 M KOH	68	86	[14]
Co@N-CNT	1.0 M KOH	74	84	[15]
MoO_3/Ni-NiO	1.0 M KOH	62	59	[16]
CoFeZr oxides/NF	1.0 M KOH	104	54.2	[17]
CoMoNiS	1.0 M KOH	113	85	[18]
Mo-NiO/Ni	1.0 M KOH	50	86	[19]
NiCoP	1.0 M KOH	130	83	[20]
Ni-FeP/TiN	1.0 M KOH	75	73	[21]
$Ni(OH)_2$/CuS	1.0 M KOH	95	104	[22]
C_3N_4@MoN	1.0 M KOH	110	57.8	[23]
Co@$CoMoO_4$	1.0 M KOH	46	85	[24]
$NiMoO_4$@NiS_2	1.0 M KOH	99	74.2	[25]
Co/β-Mo_2C@N-CNTs	1.0 M KOH	170	92	[26]

Ni_5Co_3Mo-OH	1.0 M KOH	52	59	[27]
NiMoN/CFC	1.0 M KOH	40	70	[28]
Co_3Mo/MoO_x/Ni	1.0 M KOH	68	61	[29]
o-$CoSe_2P$	1.0 M KOH	104	69	[30]
Ni-FeP/C	1.0 M KOH	95	72	[31]
$NiFeO_x$@NiCu	1.0 M KOH	70	68	[32]
Co_3S_4/MOF	1.0 M KOH	80	82	[33]
Mo-Co_9S_8@C	1.0 M KOH	113	68	[34]

Table S2: Comparison of the OER activity of Co/CoMoN/NF with other non-noble metal based electrocatalysts in alkaline electrolytes.

Catalysts	Electrolyte	η_{10} (mV)	Tafel slope (mV/dec)	Reference
Co/CoMoN/NF	1.0 M KOH	248	56	*This work*
N-Ni_3S_2	1.0 M KOH	300	70	[35]
NiOOH/Ni_5P_4	1.0 M KOH	290	40	[36]
$NiCo_2O_4$	1.0 M KOH	420	90	[37]
NiFeCo-LDH/CF	1.0 M KOH	249	42	[38]
NiFe-MoO_x NS	1.0 M KOH	276	55	[39]
Ni-MoN	1.0 M KOH	276	98	[40]
Fe_3N/Fe_4N	1.0 M KOH	238	44.5	[41]
Ni/NiFeMoO_x/NF	1.0 M KOH	255	35	[42]
Fe-CoP/CoO	1.0 M KOH	219	52	[43]
Co/VN	1.0 M KOH	320	55	[44]
Ni_2Co-N	1.0 M KOH	214	53	[45]
Ni_3FeN	1.0 M KOH	259	54	[46]
CoV hydro(oxy)oxide	1.0 M KOH	250	44	[47]
Ag/$Co(OH)_x$	1.0 M KOH	250	76	[48]
Fe/CoOOH/graphene	1.0 M KOH	330	37	[49]
$Fe_xCo_{1-x}OOH$	1.0 M KOH	266	30	[50]
$Co_{0.75}Ni_{0.25}(OH)_2$	1.0 M KOH	235	56	[51]

γ-CoOOH nanosheets	1.0 M KOH	275	49	[52]
F-Co_3Fe LDH	1.0 M KOH	287	39.2	[53]

Table S3: Comparison of the overall water splitting activity of Co/CoMoN/NF with other non-noble metal based electrocatalysts in alkaline electrolytes.

Catalysts	J (mA cm^{-2})	Potential (V)	Reference
Co/CoMoN/NF	10	1.50	This work
Mo-Ni_3S_2/Ni_xP_y/NF	10	1.46	[54]
Mo-NiP_x/NiS_y	10	1.42	[55]
Ni_2P-Ni_3S_2 HNAs/NF	10	1.50	[56]
O-CoMoS	10	1.60	[57]
$NiMoO_x$/NiMoS	10	1.46	[58]
$Ni_{0.51}Co_{0.49}P$	10	1.57	[59]
NiFe LDH/Cu NW	10	1.54	[60]
Fe-CoP/$Ni(OH)_2$	10	1.52	[61]
Co_4Ni_1P nanotubes	10	1.59	[62]
$Ni_{0.33}Co_{0.67}MoS_4$	10	1.55	[63]
CoP@NiFe-OH/SP	10	1.53	[64]
V-CoP@a-CeO_2	10	1.56	[65]
$Co(OH)_2$/NiMo CA	10	1.52	[66]
CoNC@Co_2N/CPs	10	1.52	[67]
Ni/$NiFeMoO_x$	10	1.50	[42]
$NiCo_2S_4$	10	1.58	[68]
CoSAs-MoS_2/TiN NRs	10	1.65	[69]
Ni/γ-Fe_2O_3	10	1.47	[70]

FeCoNi nanosheets/CC	10	1.55	[71]
NiMoO	10	1.54	[72]
FeCoNi nanotubes	10	1.43	[73]
CoP/NC	10	1.64	[74]
NiCoP@NC	10	1.58	[75]
NiMoN/CFC	10	1.64	[28]
Mo-Co_9S_8@C	10	1.56	[34]

References

[1] X. Zou, Y. Wu, Y. Liu, D. Liu, W. Li, L. Gu, H. Liu, P. Wang, L. Sun, Y. Zhang, *Chem* **2018**, *4*, 1–14.

[2] K. Zhu, J. Chen, W. Wang, J. Liao, J. Dong, M. O. L. Chee, N. Wang, P. Dong, P. M. Ajayan, S. Gao, J. Shen, M. Ye, *Adv. Funct. Mater.* **2020**, 2003556.

[3] J. Lv, P. Liu, R. Li, L. Wang, K. Zhang, P. Zhou, X. Huang, G. Wang, *Appl. Catal. B: Environ.* **2021**, *298*, 120587.

[4] H. Huang, H. Jung, S. Li, S. Kim, J. W. Han, J. Lee, *Nano Energy* **2022**, *92*, 106763.

[5] G. Kresse, J. Furthmüller, *Phys. Rev. B*. **1996**, *54*, 11169-11186.

[6] P. E. Blöchl, *Phys. Rev. B*. **1994**, *50*, 17953-17979.

[7] J. P. Perdew, K. Burke, M. Ernzerhof, *Phys. Rev. Lett.* **1996**, *77*, 3865.

[8] S. Grimme, J. Antony, S. Ehrlich, H. Krieg, *J. Chem. Phys*. **2010**. *132*, 154104.

[9] W. Tang, E. Sanville, G. Henkelman, *J. Phys. Condens. Matter*. **2009**, *21*, 084204.

[10] G. Henkelman, B. P. Uberuaga, H. Jónsson, *The Journal of chemical physics*. **2000**, *113*, 9901-9904.

[11] L. Dai, Z. N. Chen, L. Li, P. Yin, Z. Liu, H. Zhang, *Adv. Mater*. **2020**, *32*, 1906915.

[12] H. L. S. Santos, P. G. Corradini, M. Medina, J. A. Dias, L. H. Mascaro, *ACS Appl. Mater. Interfaces* **2020**, *12*, 17492-17501.

[13] X. Li, L. Xiao, L. Zhou, Q. Xu, J. Weng, J. Xu, B. Liu, *Angew. Chem., Int. Ed.* **2020**, *59*, 21106-21113.

[14] C. Huang, L. Yu, W. Zhang, Q. Xiao, J. Zhou, Y. Zhang, P. An, J. Zhang, Y. Yu, *Appl. Catal. B: Environ.* **2020**, *276*, 119137.

[15] L. Yang, H. Li, Y. Yu, Y. Wu, L. Zhang, *Appl. Catal. B: Environ.* **2020**, *271*, 118939.

[16] X. Li, Y. Wang, J. Wang, Y. Da, J. Zhang, L. Li, C. Zhong, Y. Deng, X. Han, W. Hu, *Adv. Mater.* **2020**, *32*, 2003414.

[17] L. Huang, D. Chen, G. Luo, Y. R. Lu, C. Chen, Y. Zou, C. L. Dong, Y. Li, S. Wang, *Adv. Mater.* **2019**, *31*, 1901439.

[18] Y. Yang, H. Yao, Z. Yu, S. M. Islam, H. He, M. Yuan, Y. Yue, K. Xu, W. Hao, G. Sun, H. Li, S. Ma, P. Zapol, M. G. Kanatzidis, *J. Am. Chem. Soc.* **2019**, *141*, 10417-10430.

[19] J. Huang, J. Han, T. Wu, K. Feng, T. Yao, X. Wang, S. Liu, J. Zhong, Z. Zhang, Y. Zhang, B. Song, *ACS Energy Lett.* **2019**, *4*, 3002-3010.

[20] S. Surendran, S. Shanmugapriya, A. Sivanantham, S. Shanmugam, R. K. Selvan, *Adv. Energy Mater.* **2018**, *8*, 1800555.

[21] X. Peng, A. M. Qasim, W. Jin, L. Wang, L. Hu, Y. Miao, W. Li, Y. Li, Z. Liu, K. Huo, K. Wong, P. K. Chu, *Nano Energy* **2018**, *53*, 66-73.

[22] S. Q. Liu, H. R. Wen, Y. Guo, Y. W. Zhu, X. Z. Fu, R. Sun, C. P. Wong, *Nano Energy* **2018**, *44*, 7–14.

[23] H. Jin, X. Liu, Y. Jiao, A. Vasileff, Y. Zheng, S. Z. Qiao, *Nano Energy* **2018**, *53*, 690–697.

[24] R. Xiang, Y. Duan, L. Peng, Y. Wang, C. Tong, L. Zhang, Z. Wei, *Appl. Catal. B Environ.* **2019**, *246*, 41–49.

[25] L. An, J. Feng, Y. Zhang, R. Wang, H. Liu, G. C. Wang, F. Cheng, P. Xi, *Adv. Funct. Mater.* **2019**, 29, 1805298.

[26] T. Ouyang, Y. Q. Ye, C. Y. Wu, K. Xiao, Z. Q. Liu, *Angew. Chem., Int. Ed.* **2019**, *58*, 4923–4928.

[27] S. Hao, L. Chen, C. Yu, B. Yang, Z. Li, Y. Hou, L. Lei, X. Zhang, *ACS Energy Lett.* **2019**, *4*, 952-959.

[28] Y. Li, X. Wei, L. Chen, J. Shi, M. He, *Nat. Commun.* **2019**, *10*, 5335.

[29] J. Chen, Y. Ge, Q. Feng, P. Zhuang, H. Chu, Y. Cao, W. R. Smith, P. Dong, M. Ye, J. Shen, *ACS Appl. Mater. Interface* **2019**, *11*, 9002-9010.

[30] Y. R. Zheng, P. Wu, M. R. Gao, X. L. Zhang, F. Y. Gao, H. X. Ju, R. Wu, Q. Gao, R. You, W. X. Huang, S. J. Liu, S. W. Hu, J. Zhu, Z. Li, S. H. Yu, *Nat. Commun.* **2018**, *9*, 2533.

[31] X. F. Lu, L. Yu, X. W. Lou, *Sci. Adv.* **2019**, *5*, eaav6009.

[32] Y. Zhou, Z. Wang, Z. Pan, L. Liu, J. Xi, X. Luo, Y. Shen, *Adv. Mater.* **2019**, *31*, 1806769.

[33] T. Liu, P. Li, N. Yao, T. Kong, G. Cheng, S. Chen, W. Luo, *Adv. Mater.* **2019**, *31*, 1806672.

[34] L. Wang, X. Duan, X. Liu, J. Gu, R. Si, Y. Qiu, Y. Qiu, D. Shi, F. Chen, X. Sun, J. Lin, J. Sun, *Adv. Energy Mater.* **2020**, *10*, 1903137.

[35] P. Chen, T. Zhou, M. Zhang, Y. Tong, C. Zhong, N. Zhang, L. Zhang, C. Wu, Y. Xie, *Adv. Mater.* **2017**, *29*, 1701584.

[36] M. Ledendecker, S. K. Calderón, C. Papp, H. P. Steinrück, M. Antonietti, M. Shalom, *Angew. Chem., Int. Ed.* **2015**, *54*, 12361-12365.

[37] X. Yu, Z. Sun, Z. Yan, B. Xiang, X. Liu, P. Du, *J. Mater. Chem. A.* **2014**, *2*, 20823-20831.

[38] Y. Lin, H. Wang, C. K. Peng, L. Bu, C. L. Chiang, K. Tian, Y. Zhao, J. Zhao, Y. G. Lin, J. M. Lee, L. Gao, *Small* **2020**, *16*, 2002426.

[39] C. Xie, Y. Wang, K. Hu, L. Tao, X. Huang, J. Huo, S. Wang, *J. Mater. Chem. A.* **2017**, *5*, 87-91.

[40] C. Zhu, Z. Yin, W. Lai, Y. Sun, L. Liu, X. Zhang, Y. Chen, S. L. Chou, *Adv. Energy Mater.* **2018**, *8*, 1802327.

[41] F. Yu, H. Zhou, Z. Zhu, J. Sun, R. He, J. Bao, S. Chen, Z. Ren, *ACS Catal.* **2017**, *7*, 2052-2057.

[42] Y. K. Li, G. Zhang, W. T. Lu, F. F. Cao, *Adv. Sci.* **2020**, *7*, 1902034.

[43] X. Hu, S. Zhang, J. Sun, L. Yu, X. Qian, R. Hu, Y. Wang, H. Zhao, J. Zhu, *Nano Energy* **2019**, *56*, 109-117.

[44] X. Peng, L. Wang, L. Hu, Y. Li, B. Gao, H. Song, C. Huang, X. Zhang, J. Fu, K. Huo, P. K. Chu, *Nano Energy* **2017**, *34*, 1-7.

[45] X. Gao, Y. Yu, Q. Liang, Y. Pang, L. Miao, X. Liu, Z. Kou, J. He, S. J. Pennycook, S. Mu, J. Wang, *Appl. Catal. B: Environ.* **2020**, *270*, 118889.

[46] A. Saad, H. Shen, Z. Cheng, R. Arbi, B. Guo, L. S. Hui, K. Liang, S. Liu, J. P. Attfield, A. Turak, J. Wang, M. Yang, *Nano-Micro Letters* **2020**, *12*, 79.

[47] J. Liu, Y. Ji, J. Nai, X. Niu, Y. Luo, L. Guo, S. Yang, *Energy Environ. Sci.* **2018**, *11*, 1736-1741.

[48] Z. Zhang, X. Li, C. Zhong, N. Zhao, Y. Deng, X. Han, W. Hu, *Angew. Chem., Int. Ed.* **2020**, *59*, 7245-7250.

[49] X. Han, C. Yu, S. Zhou, C. Zhao, H. Huang, J. Yang, Z. Liu, J. Zhao, J. Qiu, *Adv. Energy Mater.* **2017**, *7*, 1602148.

[50] S. H. Ye, Z. X. Shi, J. X. Feng, Y. X. Tong, G. R. Li, *Angew. Chem., Int. Ed.* **2018**, *57*, 2672-2676.

[51] X. Wang, Z. Li, D. Y. Wu, G. R. Shen, C. Zou, Y. Feng, H. Liu, C. K. Dong, X. W. Du, *Small* **2019**, *15*, 1804832.

[52] H. Wang, E. Feng, Y. Liu, C. Zhang, *J. Mater. Chem. A.* **2019**, *7*, 7777-7783.

[53] Z. Liu, C. L. Dong, Y. C. Huang, J. Cen, H. Yang, X. Chen, X. Tong, D. Su, Y. Wang, S. Wang, *J. Mater. Chem. A.* **2019**, *7*, 14483-14488.

[54] X. Luo, P. Ji, P. Wang, R. Cheng, D. Chen, C. Lin, J. Zhang, J. He, Z. Shi, N. Li, S. Xiao, S. Mu, *Adv. Energy. Mater.* **2020**, *10*, 1903891.

[55] J. Wang, M. Zhang, G. Yang, W. Song, W. Zhong, X. Wang, M. Wang, T. Sun, Y. Tang, *Adv. Funct. Mater.* **2021**, 2101532.

[56] L. Zeng, K. Sun, X. Wang, Y. Liu, Y. Pan, Z. Liu, D. Cao, Y. Song, S. Liu, C. Liu, *Nano Energy* **2018**, *51*, 26-36.

[57] J. Hou, B. Zhang, Z. Li, S. Cao, Y. Sun, Y. Wu, Z. Gao, L. Sun, *ACS Catal.* **2018**, *8*, 4612-4621.

[58] P. Zhai, Y. Zhang, Y. Wu, J. Gao, B. Zhang, S. Cao, Y. Zhang, Z. Li, L. Sun, J. Hou, *Nat. Commun.* **2020**, *11*, 5462.

[59] J. Yu, Q. Li, Y. Li, C. Y. Xu, L. Zhen, V. P. Dravid, J. Wu, *Adv. Funct. Mater.* **2016**, *26*, 7644-7651.

[60] L. Yu, H. Zhou, J. Sun, F. Qin, F. Yu, J. Bao, Y. Yu, S. Chen, Z. Ren, *Energy Environ. Sci.* **2017**, *10*, 1820-1827.

[61] X. Yu, J. Zhao, M. Johnsson, *Adv. Funct. Mater.* **2021**, 2101578.

[62] L. Yan, L. Cao, P. Dai, X. Gu, D. Liu, L. Li, Y. Wang, X. Zhao, *Adv. Funct. Mater.* **2017**, *27,* 1703455.

[63] L. Hang, T. Zhang, Y. Sun, D. Men, X. Lyu, Q. Zhang, W. Cai, Y. Li, *J. Mater. Chem. A.* **2018**, *6*, 19555-19562.

[64] Y. Li, S. Guo, T. Jin, Y. Wang, F. Cheng, L. Jiao, *Nano Energy* **2019**, *63*, 103821.

[65] L. Yang, R. Liu, L. Jiao, *Adv. Funct. Mater*. **2020**, 1909618.

[66] Q. Zhang, W. Xiao, W. H. Guo, Y. X. Yang, J. L. Lei, H. Q. Luo, N. B. Li, *Adv. Funct. Mater*. **2021**, 2102117.

[67] J. Jiang, P. Yan, Y. Zhou, Z. Cheng, X. Cui, Y. Ge, Q. Xu, *Adv. Energy Mater.* **2020**, *10*, 2002214.

[68] Z. Kang, H. Guo, J. Wu, X. Sun, Z. Zhang, Q. Liao, S. Zhang, H. Si, P. Wu, L. Wang, Y. Zhang, *Adv. Funct. Mater*. **2019**, *29*, 1807031.

[69] T. L. L. Doan, D. C. Nguyen, S. Prabhakaran, D. H. Kim, D. T. Tran, N. H. Kim, J. H. Lee, *Adv. Funct. Mater.* **2021**, 2100233.

[70] B. H. R. Suryanto, Y. Wang, R. K. Hocking, W. Adamson, C. Zhao, *Nat. Commun.* **2019**, *10*, 5599.

[71] Q. Zhang, N. M. Bedford, J. Pan, X. Lu, R. Amal, *Adv. Energy Mater*. **2019**, *9*, 1901312.

[72] Z. Y. Yu, C. C. Lang, M. R. Gao, Y. Chen, Q. Q. Fu, Y. Duan, S. H. Yu, *Energy Environ. Sci*. **2018**, *11*, 1890-1897.

[73] H. Li, S. Chen, Y. Zhang, Q. Zhang, X. Jia, Q. Zhang, L. Gu, X. Sun, L. Song, X. Wang, *Nat. Commun.* **2018**, *9*, 2452.

[74] Y. Pan, K. Sun, S. Liu, X. Cao, K. Wu, W. C. Cheong, Z. Chen, Y. Wang, Y. Li, Y. Liu, D. Wang, Q. Peng, C. Chen, Y. Li, *J. Am. Chem. Soc.* **2018**, *140*, 2610-2618.

[75] B. Cao, Y. Cheng, M. Hu, P. Jing, Z. Ma, B. Liu, R. Gao, J. Zhang, *Adv. Funct. Mater.* **2019**, *29*, 1906316.
